# One-pot non-enzymatic formation of firefly luciferin in a neutral buffer from *p*-benzoquinone and cysteine

**DOI:** 10.1038/srep24794

**Published:** 2016-04-21

**Authors:** Shusei Kanie, Toshio Nishikawa, Makoto Ojika, Yuichi Oba

**Affiliations:** 1 Graduate School of Bioagricultural Sciences, Nagoya University, Nagoya, 464-8601, Japan; 2 Department of Environmental Biology, College of Bioscience and Biotechnology, Chubu University, Kasugai 487-8501, Japan

## Abstract

Firefly luciferin, the substrate for the bioluminescence reaction of luminous beetles, possesses a benzothiazole ring, which is rare in nature. Here, we demonstrate a novel one-pot reaction to give firefly luciferin in a neutral buffer from *p*-benzoquinone and cysteine without any synthetic reagents or enzymes. The formation of firefly luciferin was low in yield in various neutral buffers, whereas it was inhibited or completely prevented in acidic or basic buffers, in organic solvents, or under a nitrogen atmosphere. Labelling analysis of the firefly luciferin using stable isotopic cysteines showed that the benzothiazole ring was formed *via* the decarboxylation and carbon-sulfur bond rearrangement of cysteine. These findings imply that the biosynthesis of firefly luciferin can be developed/evolved from the non-enzymatic production of firefly luciferin using common primary biosynthetic units, *p*-benzoquinone and cysteine.

Firefly luciferin (D-firefly luciferin) is the substrate commonly used for the bioluminescence reaction of luminous beetles, fireflies (the family Lampyridae), railroad worms (Phengodidae), and fire beetles (Elateridae)^1^. This reaction is catalysed by firefly luciferase in the presence of ATP, Mg^2+^, and molecular oxygen^1^. This luminescence system is widely used in every field of the life sciences, such as for real-time gene expression imaging^2^.

Natural firefly luciferin was isolated from the lanterns of the North American firefly *Photinus pyralis*^3^, and the chemical structure was determined to be (*S*)-2-(6′-hydroxy-2′-benzothiazolyl)-2-thiazoline-4-carboxylic acid by chemical synthesis^4^,^5^. The (*R*)-enantiomer (L-firefly luciferin) is inactive for the bioluminescence reaction^6^,^7^. Although the benzothiazole ring is a common scaffold that can be found in a wide variety of biologically and pharmacologically active compounds^8^, it is relatively rare in nature^9^. For this reason, research efforts have been directed towards not only the development of chemical synthetic methods but also the clarification of biosynthetic processes of benzothiazole compounds^10^,^11^,^12^.

McCapra and Razavi reported the chemical synthesis of ethyl 6-hydroxybenzothiazole-2-carboxylate from *p*-benzoquinone and cysteine ethyl ester hydrochloride in three steps, and suggested that firefly luciferin was biosynthesized from *p*-benzoquinone and cysteine in nature^13^. We recently revealed that D-firefly luciferin can be biosynthesized from one molecule of hydroquinone/*p*-benzoquinone and two molecules of L-cysteine by injecting stable isotope-labeled compounds into the adult lantern of a living Japanese firefly *Luciola lateralis*^14^. We also showed that its benzothiazole ring formation was accompanied by the decarboxylation of L-cysteine^14^. However, the details of the biosynthetic process of firefly luciferin, including the intermediates and the rearrangement mechanism for benzothiazole ring formation, have remained unclear.

Studying chemistry in aqueous solution can provide valuable insights into the synthetic processes of natural products in nature, because enzymes operate in aqueous solution^15^. For example, Vilotijevic and Jamison demonstrated that epoxide-opening cascades occur in neutral water, supporting Nakanishi’s hypothesis for the biosynthesis of ladder polyethers found in various marine natural products^16^. Chapman *et al*. reported biomimetic synthesis of the natural plant-derived product, carpanone, from two molecules of carpacin using Pd(II) in aequous methanolic solution^17^,^18^. Robinson reported the one-pot synthesis of tropinone from succindialdehyde, methylamine, and acetonedicarboxylic acid in neutral aqueous solution^19^. Later studies revealed that the Robinson’s synthesis mimics the biosynthesis of tropinone^20^. Chemical synthesis in water has also recently attracted considerable attention as one of the principles of ‘green chemistry’^21^, and there have been numerous reports on the successful synthesis of various bioactive compounds based on this concept^22^.

In this study, we demonstrate a novel one-pot reaction to give firefly luciferin in a neutral buffer from *p*-benzoquinone and cysteine without any synthetic reagents or enzymes. We also show that the benzothiazole ring of the firefly luciferin is formed *via* the decarboxylation and carbon-sulfur rearrangement of cysteine in the one-pot reaction.

## Results

### One-pot formation of firefly luciferin from *p*-benzoquinone and L-cysteine under a neutral buffer condition

**Figure 1:**
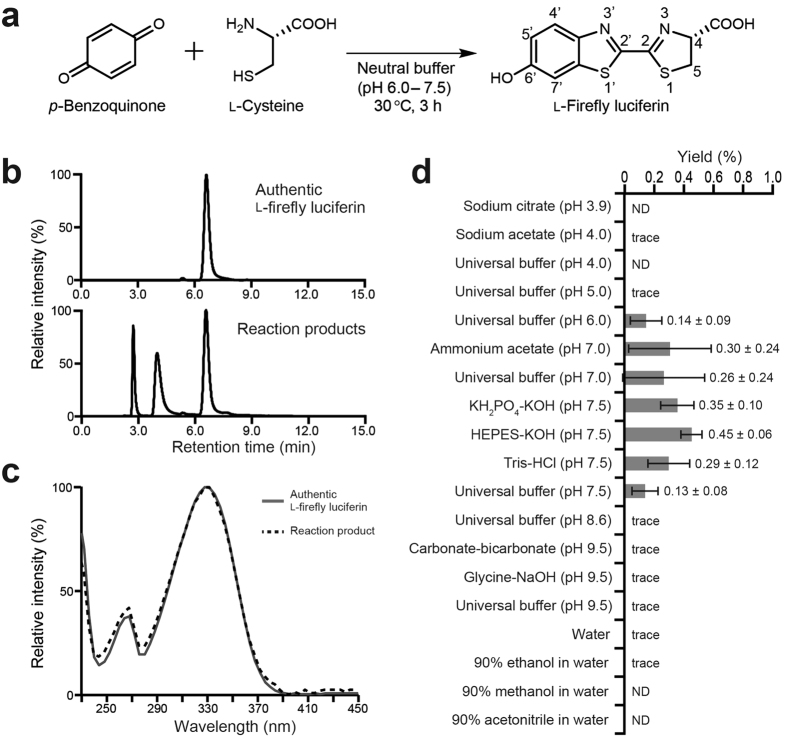
Formation of firefly luciferin from *p*-benzoquinone and L-cysteine. (**a**) Scheme for the reaction of *p*-benzoquinone with L-cysteine to give L-firefly luciferin under various neutral buffer conditions. (**b**) HPLC analysis of the products obtained by the reaction of *p*-benzoquinone with L-cysteine in 90 mM Tris-HCl (pH 7.5) using a fluorescence detector. (**c**) Absorption spectrum of the reaction product obtained at a retention time of 6.5 min, corresponding to the peak of authentic L-firefly luciferin. (**d**) Yields of L-firefly luciferin from the reaction of *p*-benzoquinone with L-cysteine in various solvents (Supplementary Table S2, entries 1–12, 29–35). The yields based on cysteine were determined by HPLC analysis against a calibration curve (Supplementary Fig. S3). ND and trace indicate “not detected” and “undetectable levels of yield <0.003%”, respectively. The pH value of the reaction mixture in water was 5.0 because of the acidity of cysteine. The bars represent the mean values ± S.D. for four replicate experiments.

Using HPLC with a UV-visible detector, we found a product corresponding to authentic firefly luciferin in the reaction mixture of *p*-benzoquinone and L-cysteine (1:1) in Tris-HCl (pH 7.5) (Fig. 1b,c). The product was identified as firefly luciferin by NMR analysis (Supplementary Fig. S1 and Table S1).

### Effects of reaction conditions on the yield of firefly luciferin

The above-described one-pot formation of firefly luciferin reached a plateau at 1 h after the start of the reaction, with further increases in the reaction time having no discernible impact on formation (Supplementary Table S2, entries 41–44). The average yields were 0.13–0.45% under various neutral buffer conditions (pH 6.0–7.5). The nature of the buffer was not essential for the formation of firefly luciferin (Fig. 1d, and Supplementary Table S2, entries 3–6, 13–18, 31–33). The production of firefly luciferin was not detected or detected in only trace amounts under acidic buffer (pH 4.0–5.0), basic buffer (pH 8.6–9.5), water, or organic solvent (methanol, ethanol, or acetonitrile) conditions (Fig. 1d, Supplementary Table S2, entries 1, 2, 7–12, 29, 30, 34, 35). The concentrations of *p*-benzoquinone and L-cysteine in the reaction mixture had no discernible impact on the yield of firefly luciferin (Supplementary Table S2, entries 45–49; Student’s t-test, *P* > 0.1 for comparisons among the entries). In contrast, the molar ratio of *p*-benzoquinone to L-cysteine had a pronounced effect on the yield (supplementary Table S2, entries 19–28): the use of an excess of L-cysteine (*p*-benzoquinone: L-cysteine = 1:2, or 1:5) did not affect the yield (entries 19, 20, 24, 25; Student’s t-test, *P* > 0.1 for comparison with entries 21, 26), whereas the use of an excess of *p*-benzoquinone (*p*-benzoquinone: L-cysteine = 2:1 or 5:1) did not result in firefly luciferin formation (undetectable levels). The reaction temperature also affected the yield: the reaction of a 1:1 mixture of *p*-benzoquinone and L-cysteine at 4 °C resulted in an average firefly luciferin yield of less than 0.003% (Supplementary Table S2, entry 36), whereas the average yields at 30 °C, 60 °C, and 90 °C were 0.30%, 0.70%, and 0.22%, respectively (Supplementary Table S2, entries 3, 37–38).

### Oxygen requirement for the formation of firefly luciferin

There was a considerable decrease in the yield of firefly luciferin (less than 0.005%) when the one-pot reaction was conducted under a nitrogen atmosphere (Supplementary Table S2, entry 39). In contrast, the use of an oxygen atmosphere did not improve the yield (Supplementary Table S2, entry 40; Student’s t-test, *P* > 0.1 for comparison with entry 3).

### Enantioselectivity of firefly luciferin formed in the one-pot reaction

**Figure 2:**
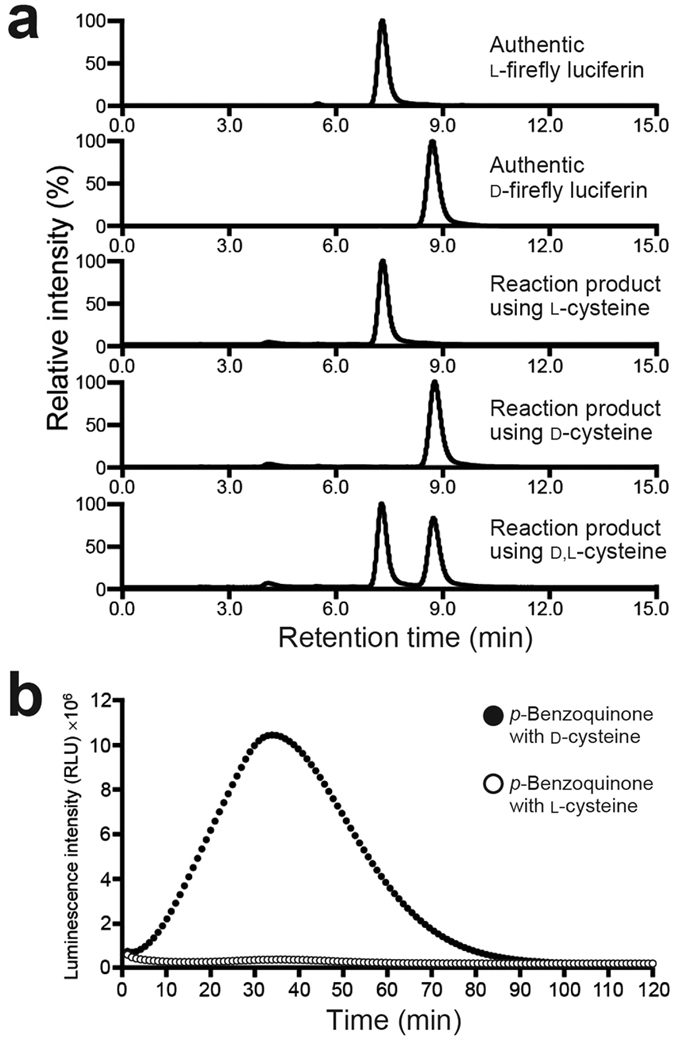
Enantioselective formation of firefly luciferin from *p*-benzoquinone and D-cysteine or L-cysteine. (**a**) Chiral HPLC analysis of the reaction products formed from *p*-benzoquinone and D-cysteine or L-cysteine. (**b**) Luminescence of a mixture of *p*-benzoquinone and D-cysteine or L-cysteine in the presence of ATP, Mg^2+^, and firefly luciferase.

HPLC analysis using a chiral column showed that firefly luciferin produced by the one-pot reaction of *p*-benzoquinone with L-cysteine was only the L-form, whereas the reaction of *p*-benzoquinone with D-cysteine was only the D-form (Fig. 2a). The formation of natural D-form firefly luciferin from *p*-benzoquinone and D-cysteine was further demonstrated by time-course luminescence monitoring in the presence of firefly luciferase, Mg^2+^, and ATP. The results showed that the luminescence intensity increased gradually and reached its maximum value at 40 min after the initial mixing of *p*-benzoquinone with D-cysteine (Fig. 2b).

### Decarboxylation of cysteine during benzothiazole ring formation

**Figure 3:**
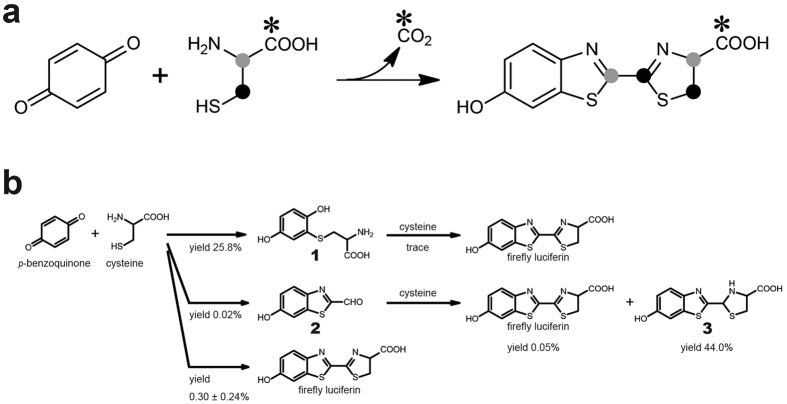
Schemes for the formation of firefly luciferin under neutral buffer conditions. (**a**) Origins of the carbons in the firefly luciferin produced by the one-pot reaction. (**b**) Products obtained by the reaction of *p*-benzoquinone with cysteine (1:1) in ammonium acetate buffer (pH 7.0). The yields of compounds **1** and **2**, as well as that of firefly luciferin (see Fig. 1d), were based on *p*-benzoquinone and cysteine, respectively. Compounds **1** and **2** were converted into firefly luciferin by the reaction with cysteine in ammonium acetate buffer (pH 7.0) in trace and 0.05% yields, respectively.

LC/ESI-TOF-MS analysis of firefly luciferin formed from *p*-benzoquinone and L-[U-^13^C_3_]-cysteine showed that one carbon atom of the cysteine was eliminated during the one-pot formation of firefly luciferin (Supplementary Fig. S2). HMBC analysis of the firefly luciferin formed by the one-pot reaction of *p*-benzoquinone with L-[1-^13^C]-cysteine showed that H-4 (δ 5.19) and H-5 (δ 3.71 and 3.75) were correlated to the carbonyl carbon (δ 177.8), indicating that the carbonyl carbon atom was the only atom enriched with ^13^C (Supplementary Fig. S4). These data therefore suggested that the C-1 carbon atom of one cysteine was eliminated during the benzothiazole ring formation of firefly luciferin (Fig. 3a).

### Origins of C-2’ and C-2 carbon atoms of firefly luciferin produced by the one-pot reaction

HMBC analysis of the firefly luciferin formed by the one-pot reaction of *p*-benzoquinone with L-[3-^13^C]-cysteine showed correlations from H-4 (δ 5.19) to C-2 (δ 165.2) and C-5 (δ 37.3), indicating that the C-2 and C-5 carbon atoms were the only carbons enriched with ^13^C (Supplementary Fig. S5). These data therefore suggested that the C-2′ and C-2 carbon atoms of the firefly luciferin were derived from the C-2 and C-3 carbon atoms of the cysteine, respectively (Fig. 3a).

### Formation of *S*-(2,5-dihydroxyphenyl)cysteine (1) and 6-hydroxybenzothiazole-2-carbaldehyde (2) from *p*-benzoquinone and L-cysteine

HPLC, NMR, and MS analyses revealed that *S*-(2,5-dihydroxyphenyl)cysteine (**1**) and 6-hydroxybenzothiazole-2-carbaldehyde (**2**), as well as firefly luciferin, were formed during the one-pot reaction of *p*-benzoquinone with L-cysteine (Supplementary Figs. S6 and S7). In this experiment, the yields of compounds **1**, **2** were 25.8% (determined by HPLC) and 0.015% (determined by NMR), respectively (Fig. 3b). Under the same reaction condition, we detected the production of hydroquinone in 24.1% yield by HPLC analysis (Supplementary Fig. S8). Under a 90 mM ammonium acetate (pH 7.0) condition for 3 h at 30 °C, the reaction of compound **2** with D-cysteine gave firefly luciferin in 0.047% yield and 2-(6′-hydroxy-2′-benzothiazolyl)-2-thiazolidine-4-carboxylic acid (**3**) in 44.0% yield (isolated yield) (Fig. 3b and Supplementary Fig S9), while the reaction of compound **1** with D-cysteine gave only a trace amount of firefly luciferin (Supplementary Fig. S10) and 94.0% recovery of compound **1**.

## Discussion

In this study, we have demonstrated a novel one-pot reaction to give firefly luciferin from *p*-benzoquinone and cysteine in a neutral buffer without any synthetic reagents or enzymes under an air atmosphere at ambient temperature. Although production occurred under various neutral buffer conditions, it was inhibited or completely prevented under acidic or basic buffer conditions, in the presence of an organic solvent, under a nitrogen atmosphere, or at lower temperature. The yields of firefly luciferin were very low and unsteady in every experiment, but the yields represented by a numerical value were at least 10 times higher than those represented by ‘ND’ or ‘trace’ (see Supplementary Table S2). Thus, the one-pot formation of firefly luciferin required neutral pH conditions and oxygen. The pH dependence of firefly luciferin formation can be attributed to the presence of free amino and carboxyl groups in cysteine. In a chemical synthetic study using unprotected amino acids in aqueous solution, Yokoyama *et al*. showed that the reaction product depends on the ionization states of amino and carboxyl groups of amino acids during the chemical reaction^23^. We do not have concrete ideas about oxygen’s role in the formation of firefly luciferin in the one-pot reaction. However, McCapra and Razavi reported that oxygen was required for the formation of 6-hydroxybenzothiazole-2-carboxamide from 7-acetyloxybenzothiazine-3-carboxamide in the presence of sodium ethoxide in dry ethanol. On the basis of this result, they suggested that the ring contraction from benzothiazine to benzothiazole occurred *via* an intermediate peroxide^13^. When our one-pot reaction was performed at 4 °C for 3 h, firefly luciferin was produced at undetectable levels, but when the resultant solution was further stirred at 30 °C for 3 h, it was detected in 0.09% yield (data not shown). This result indicates that the low reaction rate inhibited the formation at 4 °C.

Excess *p*-benzoquinone conditions (*p*-benzoquinone: cysteine = 2:1 or 5:1 mol/mol) led to a dramatic decrease in the yield of firefly luciferin, suggesting that the molar ratio of *p*-benzoquinone to cysteine was a critical processing parameter for the formation of firefly luciferin. The reaction of a 2:1 (mol/mol) mixture of *p*-benzoquinone with cysteine has been investigated previously in both water and phosphate buffer (pH 6)^24^,^25^. The results of those studies showed that the reactions gave compound **1**, quinonimines, benzothiazines, and several associated polymers, but no firefly luciferin. It was envisaged that the use of an excess of *p*-benzoquinone to cysteine would promote side reactions such as polymerization, thereby preventing the formation of firefly luciferin. In fact, under excess *p*-benzoquinone conditions, we observed that the reaction mixture became darker in colour and afforded a larger quantity of precipitates compared with the use of a 1:1 (mol/mol) condition.

NMR analysis of the firefly luciferin formed by the one-pot reaction of *p*-benzoquinone with L-[1-^13^C]-cysteine showed that decarboxylation of cysteine occurred during benzothiazole ring formation. This was consistent with our previous finding that D-firefly luciferin was biosynthesized *in vivo via* the decarboxylation of L-cysteine^14^.

NMR analysis of the firefly luciferin formed by the one-pot reaction of *p*-benzoquinone with L-[3-^13^C]-cysteine revealed that the C-2′ and C-2 carbon atoms of the firefly luciferin were derived from the C-2 and C-3 carbon atoms of cysteine, respectively. This result suggested that the benzothiazole ring of the firefly luciferin was formed through a carbon-sulfur bond rearrangement of cysteine in the one-pot reaction. It is noteworthy that similar rearrangement processes for the formation of the benzothiazole ring were shown in studies on the biosynthesis of antibiotic rifamycins^26^,^27^,^28^ and on the biomimetic oxidation of the pheomelanin precursor, 5-*S*-cysteinyldopa^29^.

We found that the one-pot reaction also gave compounds **1** and **2**. The formation of compound **1** is consistent with an earlier report that compound **1** was formed by the reaction of *p*-benzoquinone with cysteine in phosphate buffer (pH 6)^25^. That paper also described that compound **1** was further converted into benzothiazine in the presence of *p*-benzoquinone^25^. We did not identify the benzothiazine product, but detected *m/z* 166.0 and 182.0 ions, corresponding to the proton adducts of 7-hydroxybenzothiazine and 2,7-dihydroxybenzothiazine in *m/z* value, respectively, in the one-pot reaction mixture by mass spectrometry (Supplementary Fig. S11). With regard to the formation of compound **2**, it has been suggested that the benzothiazole-2-carbaldehyde structure was biosynthetically formed from the benzothiazine structure^10^,^11^,^30^. On the basis of these findings, we expected that compound **1** was converted into benzothiazine intermediates through the oxidation by *p*-benzoquinone and followed by the ring contraction to give compound **2**. Indeed, we detected hydroquinone in 24.1% yield after the one-pot reaction. It has been reported that the condensation of acetaldehyde with cysteine in water give 2-methylthiazolidine-4-carboxylic acid while retaining the original configuration of the cysteine stereocenter^31^, and that 2-thiazolines can be synthesized from 2-thiazolidines by Ru/PPh_3_ or Ru/TMEDA-catalysed oxidation with TBHP at ambient temperature^32^. These results are consistent with the fact that the original configuration of the cysteine stereocenter was retained through the formation of thiazoline moiety in the one-pot reaction (Fig. 2). Taking the previous and present results together, we tentatively proposed a reaction pathway for firefly luciferin from *p*-benzoquinone and cysteine *via* compound **1** and **2** (Supplementary Fig. S12).

We then chemically synthesized compounds **1** and **2**, and investigated their reaction with cysteine under a neutral buffer condition. The results showed that the reaction of compound **2** with cysteine gave firefly luciferin in low yield (0.05%) and compound **3** as a major product, whereas the reaction of compound **1** gave a trace signal corresponding to authentic firefly luciferin with almost quantitative recovery of compound **1** in HPLC analysis. These results therefore suggested that compounds **1** and **2** were possible intermediates for firefly luciferin formation during the one-pot reaction. However, detailed examinations will be necessary to clarify the mechanism underlying the one-pot formation of firefly luciferin.

We currently consider that compounds **1** and **2** are also the biosynthetic intermediates *in vivo*, because 1) the benzothiazole-2-carbaldehyde structure and *S*-(2,5-dihydroxyphenyl)cysteine occur in nature^11^,^33^, 2) the one-pot formation of firefly luciferin occurred under mild conditions, and 3) the decarboxylation of cysteine during the one-pot reaction was consistent with our finding in our previous *in vivo* experiment^14^. On the other hand, it has been proposed that 6-hydroxybenzothiazole-2-carboxylic acid and 2-cyano-6-hydroxybenzothiazole are biosynthetic intermediates of firefly luciferin^34^. However, in experiments on firefly tail extracts, Day *et al*. found no evidence that 6-hydroxybenzothiazole-2-carboxylic acid could act as a beetle luciferin precursor^34^. It has been known that 2-cyano-6-hydroxybenzothiazole readily reacts with cysteine under neutral aqueous conditions to give firefly luciferin^35^,^36^, and Gomi *et al*. suggested that 2-cyano-6-hydroxybenzothiazole was generated from firefly oxyluciferin by a luciferin-regenerating enzyme and used for the non-enzymatic regeneration of firefly luciferin *in vivo*^37^. However, the formation of 2-cyano-6-hydroxybenzothiazole by the one-pot reaction is unlikely in organic chemistry. Thus 2-cyano-6-hydroxybenzothiazole will not be considered an intermediate for firefly luciferin formation in the one-pot reaction.

It is noteworthy that there are two fundamental differences between this one-pot reaction and the *in vivo* biosynthetic process of firefly luciferin^14^. First, the one-pot reaction of hydroquinone (instead of *p*-benzoquinone) gave no firefly luciferin (Supplementary Table S2, entries 50 and 51), which is inconsistent with the fact that firefly luciferin is biosynthesized from hydroquinone^14^. Second, the one-pot reaction of *p*-benzoquinone with L-cysteine gave only L-form firefly luciferin (Fig. 2), whereas D-form firefly luciferin is biosynthesized from L-cysteine^14^. Hydroquinone can be converted into *p*-benzoquinone in organisms^38^,^39^. Furthermore, it has been suggested that D-firefly luciferin is generated from L-firefly luciferin *via* coenzyme A esterification by luciferase and the subsequent racemization and hydrolysis in adult fireflies^40^. Therefore the two fundamental differences indicated that our one-pot reaction represents the middle core steps of D-firefly luciferin biosynthesis: between ‘from hydroquinone to *p*-benzoquinone’ and ‘from L-firefly luciferin to D-firefly luciferin’. Our present findings suggest that firefly luciferin can form in life, because *p-*benzoquinone and cysteine are the primary organic compounds produced by various organisms, including insects^38^,^39^,^41^,^42^,^43^,^44^. We expected that the efficiency of firefly luciferin production from *p*-benzoquinone and cysteine would be very low at the early evolutional stages, but would improve by acquiring the biosynthetic enzyme(s), which catalyses middle core steps of the luciferin formation in the lineage of luminous beetles.

To date, various method for the chemical synthesis of firefly luciferin have been developed to readily access this valuable biological tool^5^,^45^,^46^,^47^,^48^,^49^,^50^. These methods require heat, inert gas, organic solvents, and various synthetic reagents, including strong reducing and oxidizing agents, bases, acids, and metal catalysts. Notably, the last step in all of these multi-step syntheses involved the condensation of 2-cyano-6-hydroxybenzothiazole with cysteine. In contrast, the reaction shown in the current study occurred under mild conditions in one-pot and did not proceed *via* 2-cyano-6-hydroxybenzothiazole. A detailed analysis of our one-pot reaction mechanism may therefore open up new horizons for the development of alternative strategies for the chemical synthesis of firefly luciferin and its analogues, although our reaction conditions cannot be applied directly to laboratory production because of the very low yield.

In conclusion, we have demonstrated for the first time the production of firefly luciferin from *p*-benzoquinone and cysteine by an aqueous one-pot approach. Furthermore, we have revealed that the benzothiazole ring of the firefly luciferin was formed *via* the decarboxylation and carbon-sulfur bond rearrangement of cysteine in the one-pot reaction. The evolutionary origin of firefly luciferase has been studied based on the molecular and biochemical analyses using luciferases and their homologues in beetles^51^, but the studies on the origin of firefly luciferin has been little studied^34^. Our present study illustrates that firefly luciferin has the potential to be readily originated in life.

## Methods

### Materials

The materials used in the present study were obtained from the following commercial suppliers: L-cysteine, *p*-benzoquinone (Kanto Chemical, Tokyo, Japan); D-cysteine, L-cysteine ethyl ester hydrochloride (Sigma-Aldrich, St. Louis, MO, USA); D-firefly luciferin (Wako Pure Chemical Industries, Osaka, Japan); hydroquinone (Nacalai Tesque, Kyoto, Japan); ATP (Oriental Yeast, Osaka, Japan); and recombinant *Photinus pyralis* luciferase QuantiLum (Promega, Madison, WI, USA). L-[U-^13^C_3_]-cysteine (98% isotopic purity), L-[1-^13^C]-cysteine (99% isotopic purity), L-[3-^13^C]-cysteine (99% isotopic purity) were purchased from Cambridge Isotope Laboratories (Andover, MA, USA). The chemical purities of these isotopic compounds were over 98%. L-Firefly luciferin was kindly provided by Dr Yoshiaki Toya (Aichi Univ. of Education, Aichi, Japan). All of the materials were used without further purification. It is noteworthy that the L-cysteine and *p*-benzoquinone solutions were both prepared immediately prior to their use in the following experiments.

### Chiral HPLC analysis

Chiral HPLC analysis was performed on a PU-1580 HPLC system (Jasco, Tokyo, Japan) equipped with a chiral column, CHIRALCEL OD-RH (ϕ 4.6 × 150 mm; Daicel Chemical Industry, Tokyo, Japan), a multiwavelength detector (MD-2018 Plus, Jasco), and a fluorescence detector (FP-1520, Jasco). The conditions used for the elution of the HPLC system were as follows: mobile phase, 27% (v/v) acetonitrile in H_2_O containing 0.1% (v/v) formic acid; flow rate 1.0 mL/min; fluorescence detection, excitation/emission, 330/530 nm.

### Quantification of firefly luciferin produced by the one-pot reaction in various solvents

The buffers and solvents used for the analyses were as follows: 100 mM sodium citrate buffer (pH 3.9), sodium acetate buffer (pH 4.0), universal buffer (Britton-Robinson buffer^52^) (pH 4.0, 5.0, 6.0, 7.0, 7.5, 8.6, 9.5), ammonium acetate (pH 7.0), KH_2_PO_4_-KOH (pH 7.5), HEPES-KOH (pH 7.5), Tris-HCl (pH 7.5), carbonate-bicarbonate buffer (pH 9.5), glycine-NaOH (pH 9.5), water, ethanol, methanol, and acetonitrile. To 90 μL of buffer/solvent in a 1.5 mL micro tube were added 5 μL of an 80 mM aqueous solution of L-cysteine and 5 μL of an 80 mM aqueous solution of *p*-benzoquinone. The mixture was stirred by a micro mixer (model E-36; Taitec, Saitama, Japan) in high speed mode for 3 h at 30 °C. For every reaction, we confirmed that the pH of the reaction mixture remained unchanged after the reaction by using pH test paper (Toyo Roshi, Tokyo, Japan). The resulting solution was diluted with 400 μL of H_2_O and acidified with 10 μL of 3 M HCl to below pH 3. The acidified solution was extracted with ethyl acetate (500 μL × 2). The combined organic layer was concentrated to dryness under a nitrogen stream at room temperature and dissolved in 100 μL of water by using a sonicator (model UT-206; Sharp, Osaka, Japan). The aqueous solution was centrifuged at 17,400 *× g* for 3 min at 4 °C, and 10 μL of the supernatant was subjected to chiral HPLC analysis. The absorption spectra of firefly luciferin were obtained using a multiwavelength detector.

### Luminescence monitoring of the mixture of *p*-benzoquinone and cysteine by luciferin–luciferase reaction

Immediately before this experiment, a solution of D-cysteine (or L-cysteine) in Tris-HCl (pH 7.5) was mixed with aqueous solutions of ATP and MgCl_2_. To the mixture were added *p*-benzoquinone aqueous solution and firefly luciferase in Tris-HCl (pH 7.5), followed by immediate luminescence monitoring using a Centro LB960 luminometer (Berthold, Bad Wildbad, Germany) for 21,600 s at 60 s intervals. The final mixture (110 μL) contained 4 mM *p*-benzoquinone, 4 mM D-cysteine (or L-cysteine), 2 mM ATP, 10 mM MgCl_2_, and 1.27 μg of recombinant *Photinus pyralis* luciferase in 82 mM Tris-HCl (pH 7.5).

### NMR analysis of firefly luciferin formed from *p*-benzoquinone and L-[1-^13^C]-cysteine

To 500 μL of an 80 mM solution of L-[1-^13^C]-cysteine in 100 mM Tris-HCl (pH 7.5) in a 1.5 mL micro tube was added a single portion 500 μL of an 80 mM aqueous solution of *p*-benzoquinone. After being stirred by a micro mixer (model E-36; Taitec) in high speed mode for 3 h at 30 °C, the resultant solution was washed with ethyl acetate (1 mL × 1) and the aqueous layer was acidified with 12 μL of 3 M HCl to below pH 3. The acidified solution was extracted with ethyl acetate (1 mL × 1). The organic layer was concentrated to dryness under a nitrogen stream at room temperature and dissolved in 100 μL of methanol. All of the solution was filtered through an Ultrafree-MC centrifugal filter (0.45 μm; Millipore, Billerica, MA, USA). All of the filtrate was subjected to HPLC separation with a Develosil ODS-UG-5 column (ϕ 4.6 × 250 mM; Nomura Chemical, Aichi, Japan), a multiwavelength detector (MD-2010 Plus, Jasco), and a fluorescence detector (FP-1520, Jasco). The HPLC conditions were as follows: mobile phase, linear gradient of methanol in H_2_O containing 0.1% formic acid from 10 to 100% for 45 min; flow rate 0.6 mL/min; UV detection, 327 nm; fluorescence detection, excitation/emission, 330/530 nm. The fraction eluted at a retention time of 35.5 to 37.0 min was concentrated to dryness under a nitrogen stream at room temperature and further dried *in vacuo*. The residue (containing approximately 4 μg of firefly luciferin estimated by UV absorption) was dissolved in CD_3_OD, and the solution was analysed by using an AVANCE III HD 600 Cryo-Probe NMR spectrometer (Bruker, Billerica, MA, USA).

### NMR analysis of firefly luciferin formed from *p*-benzoquinone with L-[3-^13^C]-cysteine

To 500 μL of an 80 mM solution of L-[3-^13^C]-cysteine in 100 mM Tris-HCl (pH 7.5) in a 1.5 mL micro tube was added a single portion 500 μL of an 80 mM aqueous solution of *p*-benzoquinone. After being stirred by a micro mixer (model E-36; Taitec) in high speed mode for 3 h at 30 °C, the resultant solution was separated by using an HPLC system according to the method described in the previous section. The fraction eluted at a retention time of 34.4 to 36.4 min was concentrated to dryness under a nitrogen stream at room temperature and further dried *in vacuo*. The residue (containing approximately 12 μg of firefly luciferin estimated by UV absorption) was dissolved in CD_3_OD and the solution was analysed by using an AVANCE III HD 600 Cryo-Probe NMR spectrometer (Bruker).

### Identification of compounds 1 and 2 produced by the one-pot reaction

To 50 mL of a 40 mM solution of L-cysteine in 180 mM ammonium acetate (pH 7.0) in a 500 mL Erlenmeyer flask was added a single portion 50 mL of a 40 mM aqueous solution of *p*-benzoquinone. The same reaction was also prepared in a separate flask. After being stirred (180 rpm) by a Bioshaker (model BP-98; TIC, Saitama, Japan) for 3 h at 30 °C, the resultant solutions obtained from both reactions were combined and washed with diethyl ether (150 mL × 2) and separated with ethyl acetate (200 mL × 2). To identify compound **1**, 10 μL of the aqueous layer was subjected to chiral HPLC analysis. The fraction eluted at a retention time of 11 to 12 min was subjected to HRMS analysis. To identify firefly luciferin, 100 μL of the aqueous layer was diluted with 400 μL of H_2_O and acidified with 10 μL of 3 M HCl to below pH 3. The acidified solution was extracted with ethyl acetate (500 μL × 2). The organic layer was concentrated to dryness under a nitrogen stream at room temperature and dissolved in 100 μL of H_2_O by a sonicator (model UT-206; Sharp). The aqueous solution was centrifuged at 17,400 ×* g* for 3 min at 4 °C, and 10 μL of the supernatant was subjected to chiral HPLC analysis. To identify compound **2**, 400 mL of the first obtained ethyl acetate layer was washed with brine and dried over anhydrous Na_2_SO_4_. The solution was concentrated to dryness using a rotary evaporator and further dried *in vacuo*. The residue was purified by column chromatography (silica gel 5 g; hexane-ethyl acetate, 2:1 – v/v). The purified residue was further purified by column chromatography (silica gel 5 g; dichloromethane-methanol, 98:2 – v/v). The purified product (2.2 mg) was subjected to ^1^H NMR and HRMS analyses.

## Additional Information

**How to cite this article**: Kanie, S. *et al*. One-pot non-enzymatic formation of firefly luciferin in a neutral buffer from *p*-benzoquinone and cysteine. *Sci. Rep.* **6**, 24794; doi: 10.1038/srep24794 (2016).

## Supplementary Material

Supplementary Information
