# Supplementary material for: One-pot non-enzymatic formation of firefly luciferin in a neutral buffer from p-benzoquinone and cysteine
Source: Sci Rep. 2016 Apr 21;6:24794. doi: 10.1038/srep24794 (PMC4838837; doi:10.1038/srep24794)
Supplement: Supplementary Information [file srep24794-s1.pdf]

Supplementary Information

One-pot non-enzymatic formation of firefly luciferin in a neutral buffer from *p*-benzoquinone and cysteine

Shusei Kanie, Toshio Nishikawa, Makoto Ojika & Yuichi Oba

Graduate School of Bioagricultural Sciences, Nagoya University, Nagoya, 464-8601, Japan.

Correspondence and requests for materials should be addressed to Y.O. (email: oba@agr.nagoya-u.ac.jp).

Index

1. **Supplementary Methods** **page S3**
2. **Supplementary Figures** **page S12**
3. **Supplementary Tables** **page S24**
4. **Supplementary References** **page S27**
5. **Spectra of synthesized Compounds** **page S28**

Supplementary Methods

General

Proton nuclear magnetic resonance (^{1}H NMR) spectra were recorded on an AVANCE-400 (400 MHz) or AVANCE III HD 600 Cryo-Probe (600 MHz) NMR spectrometer (Bruker, Billerica, MA, USA). The NMR chemical shifts (ppm) were referenced to the residual undeuterated solvent peaks (chloroform-*d* as δ = 7.26, methanol-d_4 as δ = 3.30), the tetramethylsilane (TMS) peak, or the 3-(trimethylsilyl)propionic acid-d_4 sodium salt (TSP-d_4) peak. Data were reported as follows: chemical shift, multiplicity (s = singlet, d = doublet, t = triplet, q = quartet, m = multiplet), coupling constant, and integration. Carbon nuclear magnetic resonance (^{13}C NMR) spectra were recorded on an AVANCE-400 (100 MHz) or AVANCE III HD 600 Cryo-Probe (150 MHz) spectrometer (Bruker). The NMR chemical shifts (ppm) were referenced to the solvent peaks (chloroform-*d* as δ = 77.0, methanol-d_4 as δ = 49.0, acetone-d_6 as δ = 29.8) and the 3-(trimethylsilyl)propionic acid-d_4 sodium salt (TSP-d_4) peak. High-resolution mass spectra (HRMS) were recorded on a Mariner Biospectrometry Workstation (Applied Biosystems, Foster City, CA, USA) in the positive ESI mode, and were reported in *m/z*. Reactions were monitored by using thin layer chromatography (TLC) on 0.25 mm silica gel coated glass plates 60 F_{254} (Merck, Darmstadt, Germany). In TLC analysis, UV light (254 nm or 366 nm), 5% ethanolic phosphomolybdic acid solution, and 2.6% (v/v) *p*-anisaldehyde in H_2SO_4-AcOH-EtOH (25:12:68 – v/v/v) were used as visualizing agents. Silica gel Wakogel-C300 (Wako Pure Chemical Industries, Osaka, Japan) was used for open-column chromatography.

NMR analysis of firefly luciferin formed from *p*-benzoquinone and L-cysteine

To 500 μL of an 80 mM solution of L-cysteine in 100 mM Tris-HCl (pH 7.5) in a 1.5 mL micro tube was added a single portion 500 μL of an 80 mM aqueous solution of *p*-benzoquinone. The L-cysteine and *p*-benzoquinone solutions were both prepared immediately prior to being used. After being stirred by a micro mixer (model E-36; Taitec, Saitama, Japan) in high speed mode for 3 h at 30 °C, the resultant solution was washed with ethyl acetate (1 mL × 1) and the aqueous layer was acidified with 12 μL of 3 M HCl to below pH 3. The pH of the aqueous solution was confirmed by using pH test paper (Toyo Roshi, Tokyo, Japan). The acidified solution was extracted with ethyl acetate (1 mL × 1). The organic layer was concentrated to dryness under a nitrogen stream at room temperature and dissolved in 100 μL of methanol. Ninety eight microliter of the methanolic solution was filtered through an Ultrafree-MC centrifugal filter (0.45 μm; Millipore, Billerica, MA, USA). All of the filtrate

was subjected to HPLC separation with a Develosil ODS-UG-5 column (ϕ 4.6 × 250 mm; Nomura Chemical, Aichi, Japan), a multiwavelength detector (MD-2010 Plus, Jasco), and a fluorescence detector (FP-1520, Jasco). The HPLC conditions were as follows: mobile phase, linear gradient of methanol in water containing 0.1% (v/v) formic acid from 10 to 100% for 45 min; flow rate 0.6 mL/min; UV detection, 327 nm; fluorescence detection, excitation/emission, 330/530 nm. The fraction eluted at a retention time of 35.0 to 37.0 min was concentrated to dryness under a nitrogen stream at room temperature and further dried *in vacuo*. The residue was dissolved in CD_3OD and the solution was analyzed by using an NMR spectrometer (600 MHz) equipped with a cryogenically cooled probe.

LC/ESI-TOF-MS analysis of the one-pot reaction products from *p*-benzoquinone and L-[U-$^{13}C_3$]-cysteine

To 30 μL of 50 mM ammonium acetate (pH 7.0) in a 1.5 mL micro tube were added 5 μL of a 100 mM aqueous solution of *p*-benzoquinone and 1 μL of a 550 mM aqueous solution of L-[U-$^{13}C_3$]-cysteine. The *p*-benzoquinone solution was prepared immediately prior to being used. After being stirred by a micro mixer (model E-36; Taitec) in high speed mode for 3 h at 30 °C, the resultant solution was centrifuged at 17,400 ×*g* for 3 min at 4 °C. The supernatant was filtered through an Ultrafree-MC centrifugal filter (0.45 μm; Millipore). The filtrate was washed with *n*-hexane (30 μL × 2) and 3 μL of the aqueous layer was subjected to LC/ESI-TOF-MS analysis. LC/ESI-TOF-MS analysis was performed by electrospray ionization time-of-flight mass spectrometry (ESI-TOF-MS) on an Agilent 1100 HPLC system (Agilent Technologies, Santa Clara, CA, USA) with a Mariner Biospectrometry Workstation (Applied Biosystems). The HPLC conditions were as follows: column, Unison UK-C8 (75 × 2 mm; Imtakt, Kyoto, Japan); mobile phase, linear gradient of methanol in water containing 0.1% (v/v) formic acid from 50 to 95% for 12 min; flow rate 0.1 mL/min; sprit ratio, 1:20 (5 μL/min); nozzle potential, 250 V; ion mode, positive. The mass value was calibrated using angiotensin I (*m/z* = 324.9272 and 432.9603) and neurotensin (*m/z* = 558.3111) as external standards.

Calibration curve for quantification of firefly luciferin

Calibration curve was determined by chiral HPLC analysis of authentic D-firefly luciferin dissolved in methanol. The amount of authentic firefly luciferin was determined spectrophotometrically, using an absorption coefficient of 18,620 M^{-1} cm^{-1} at 327 nm in methanol[1].

Quantification of firefly luciferin produced by the one-pot reaction in various buffer concentrations

To 90 μL of 50, 100, 250, 500 mM ammonium acetate (pH 7.0) or Tris-HCl (pH 7.5) in a 1.5 mL micro tube was added 5 μL of an 80 mM aqueous solution of L-cysteine and 5 μL of an 80 mM aqueous solution of *p*-benzoquinone. The L-cysteine and *p*-benzoquinone solutions were both prepared immediately prior to being used. After being stirred by a micro mixer (model E-36; Taitec) in high speed mode for 3 h at 30 °C, the resultant solution was diluted with 400 μL of water and acidified with 10 or 20 μL of 3 M HCl to below pH 3. The pH of the aqueous solution was confirmed by using pH test paper (Toyo Roshi). The acidified solution was extracted with ethyl acetate (500 μL × 2). The combined organic layer was concentrated to dryness under a nitrogen stream at room temperature and dissolved in 100 μL of water by a sonicator (model UT-206; Sharp, Osaka, Japan). The aqueous solution was centrifuged at 17,400 ×*g* for 3 min at 4 °C and 10 μL of the supernatant was subjected to chiral HPLC analysis.

Quantification of firefly luciferin produced by the one-pot reaction at various temperature

To 500 μL of a stirred 8 mM solution of L-cysteine in 180 mM ammonium acetate (pH 7.0) in a glass test tube (model ST 13-100; Nichiden-Rika, Kobe, Japan) was added a single portion 500 μL of an 8 mM aqueous solution *p*-benzoquinone. The L-cysteine and *p*-benzoquinone solutions were both prepared immediately prior to being used. After being stirred for 3 h at various temperatures (4, 30, 60, 90 °C), 100 μL of the resultant solution was pretreated as described in the previous section, and 10 μL of the resultant solution was subjected to chiral HPLC analysis. Reaction temperature was controlled using an ultra-cooling reactor (UCR-150N; Techno Sigma, Okayama, Japan) or oil bath (model W-1; Nippon Rikagaku Kikai, Tokyo, Japan). The reaction without stirring was also performed at 30 °C by the same method.

Quantification of firefly luciferin produced by the one-pot reaction under various atmospheres

An 8 mM solution of L-cysteine in 180 mM ammonium acetate (pH 7.0) and an 8 mM aqueous solution of *p*-benzoquinone were both degassed by successive freeze-pump-thaw using a nitrogen gas. These solution were both prepared immediately prior to being used. To 2 mL of the L-cysteine solution was added a single portion 2 mL of the *p*-benzoquinone solution using a common syringe technique. After being stirred for 3 h at 30 °C under balloon pressure of a nitrogen gas or 95% oxygen gas, 100 μL of the resultant solution was pretreated as described in the previous section, and 10 μL of the resultant solution was subjected to chiral HPLC analysis.

Quantification of firefly luciferin produced by the one-pot reaction of *p*-benzoquinone with L-cysteine in various final concentrations of the starting materials.

To 50 μL of an 80, 40, 20, 8, 1.6 mM solution of L-cysteine in 400 mM ammonium acetate (pH 7.0) in a 1.5 mL micro tube was added a single portion 50 μL of an 80, 40, 20, 8, 1.6 mM solution of *p*-benzoquinone aqueous solution, respectively. All solutions were prepared immediately prior to being used. After being stirred by a micro mixer (model E-36; Taitec) in high speed mode for 3 h at 30 °C, 100 μL of the resultant solution was pretreated as described in the previous section, and 10 μL of the resultant solution was subjected to chiral HPLC analysis.

Quantification of hydroquinone formed by the one-pot reaction of *p*-benzoquinone with L-cysteine

To 90 μL of ammonium acetate (pH 7.0) in a 1.5 mL micro tube were added 5 μL of an 80 mM aqueous solution of L-cysteine and 5 μL of an 80 mM aqueous solution of *p*-benzoquinone. After being stirred by a micro mixer (model E-36; Taitec) in high speed mode for 3h at 30 °C, the resultant solution was centrifuged at 17,400 ×*g* for 3 min at 4 °C. The 10 μL of the supernatant was diluted with 990 μL of water, and 10 μL of the diluted solution was subjected to HPLC analysis with a Develosil ODS-UG-5 column (ϕ 4.6 × 250 mm; Nomura Chemical, Aichi, Japan), a multiwavelength detector (MD-2010 Plus, Jasco), and a fluorescence detector (FP-1520, Jasco). The HPLC conditions were as follows: mobile phase, 25% (v/v) methanol in H_2O; flow rate 0.8 mL/min; fluorescence detection, excitation/emission, 290/ 338 nm.

Reaction of compound 1 or compound 2 with D-cysteine

To 50 μL of a 10 mM solution of D-cysteine in 180 mM ammonium acetate (pH 7.0) in a 1.5 mL micro tube was added a single portion 50 μL of a 10 mM solution of compound **1** or compound **2** suspended aqueous solution, respectively. All solutions were prepared immediately prior to being used. After being stirred by a micro mixer (model E-36; Taitec) in high speed mode for 3 h at 30 °C, 100 μL of the resultant solution was pretreated as described in the previous section, and 10 μL of the resultant solution was subjected to chiral HPLC analysis.

LC/ESI-TOF-MS analysis of the one-pot reaction products from *p*-benzoquinone and L-cysteine

A 40 mM solution of L-cysteine in 100 mM ammonium acetate (pH 7.0) and a 40 mM aqueous solution of *p*-benzoquinone were both degassed by successive freeze-pump-thaw using a nitrogen gas. These solution were both prepared immediately prior to being used. To 2.2 mL of the L-cysteine

solution was added a single portion 2.2 mL of the *p*-benzoquinone solution using a common syringe technique. After being stirred for 3 h at 30 °C under balloon pressure of air, the resultant solution was acidified with 120 μL of 3 M HCl to below pH 3. The pH of the aqueous solution was confirmed by using pH test paper (Toyo Roshi). The part of acidified solution (1 mL) was extracted with 1 mL of ethyl acetate. The organic layer was concentrated to dryness under a nitrogen stream at room temperature and dissolved in 100 μL of methanol. The methanolic solution was filtered through an Ultrafree-MC centrifugal filter (0.45 μm; Millipore) and 1 μL of the filtrate was subjected to LC/ESI-TOF-MS analysis. LC/ESI-TOF-MS analysis was performed by electrospray ionization time-of-flight mass spectrometry (ESI-TOF-MS) on an Agilent 1100 HPLC system (Agilent Technologies, Santa Clara, CA, USA) with a Mariner Biospectrometry Workstation (Applied Biosystems). The HPLC conditions were as follows: column, Unison UK-C8 (75 × 2 mm; Imtakt, Kyoto, Japan); mobile phase, linear gradient of methanol in water containing 0.1% (v/v) formic acid from 50 to 100% for 39 min; flow rate 0.1 mL/min; sprit ratio, 1:20 (5 μL/min); ion mode, positive. The mass value was calibrated using angiotensin I (*m/z* = 324.9272 and 432.9603) and neurotensin (*m/z* = 558.3111) as external standards.

Chemical Synthesis

Synthesis of compound 1

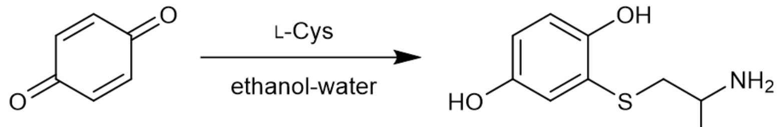

p-benzoquinone **1**

Compound **1** was prepared according to the literature[2,3]. A solution of *p*-benzoquinone (101 mg, 0.93 mmol) in ethanol (6 mL) was added a single portion to a solution of L-cysteine (150 mg, 1.24 mmol) in water (14 mL). The mixture was incubated for 1 h at room temperature. The reaction mixture was concentrated to dryness using a rotary evaporator and further dried *in vacuo*. The residue was dissolved in heated (80 °C) 50 % ethanol in water and filtered immediately through a cotton plug in a Pasteur pipette. The filtrate was cooled to room temperature and was incubated at 4 °C for 2 days to give compound **1** (129 mg, 61%) as a brownish white powder. ^{1}H NMR (400 MHz, D_2O): δ 7.05 (d, *J* = 2.8 Hz, 1H), 6.90 (d, *J* = 8.8 Hz, 1H), 6.83 (dd, *J* = 2.8, 8.8 Hz, 1H), 3.78 (dd, *J* = 4.0, 8.8 Hz, 1H), 3.48 (dd, *J* = 4.0, 14.8 Hz, 1H), 3.22 (dd, *J* = 8.8, 14.8 Hz, 1H); ^{13}C NMR (100 MHz, D_2O): δ 175.3, 152.5, 152.1, 123.5, 121.6, 120.3, 119.7, 56.5, 37.9; HRMS (*m/z*): $[M+H]^+$ calcd. for $C_9H_{12}NO_4S$, 230.04816; found, 230.04806.

Synthesis of compound 2

The synthetic route is summarized in the following scheme.

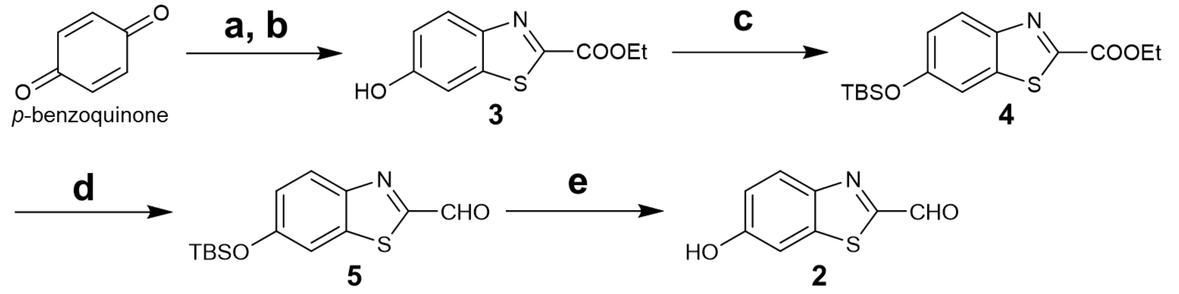

Reagents, conditions and yields: (a) L-Cys-OEt·HCl, MeOH; (b) $K_3[Fe(CN)_6]$, NaOH, *i*-PrOH, 31% in 2 steps; (c) TBSCl, imidazole, DMF, 97%; (d) DIBAL, CH_2Cl_2, -88 ℃, 46%; (e) TBAF, MeOH, 0 ℃, 73%

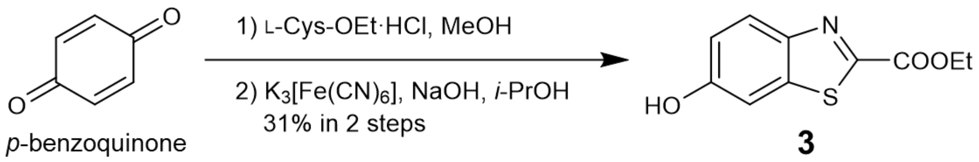

Preparation of ester 3 from *p*-benzoquinone: Ester **3** was prepared according to the literature[4-6]. A solution of *p*-benzoquinone (1.0 g, 9.2 mmol) in methanol (20 mL) was added dropwise in about 20 min to a stirred and cooled (0 °C) solution of L-cysteine ethyl ester hydrochloride (1.7 g, 9.2 mmol) in methanol (10 mL) in an ice bath under a nitrogen atmosphere. The mixture was stirred at room temperature for 1.5 h under a nitrogen atmosphere with monitoring by using TLC (hexane-ethyl acetate = 6:4 – v/v, and *n*-butanol-acetic acid-water = 6:2:2 – v/v/v). The reaction mixture was concentrated to dryness using a rotary evaporator and further dried *in vacuo*. To a stirred and cooled (0 °C) solution of the residue in isopropanol (60 mL) were added dropwise aqueous solutions of 1M $K_3[Fe(CN)_6]$ (36 mL) and 4M NaOH (2.5 mL) in the order described. The mixture was sufficiently stirred for 2.5 h at room temperature under a nitrogen atmosphere with monitoring by using TLC (hexane-ethyl acetate = 6:4 – v/v, and *n*-butanol-acetic acid-water = 6:2:2 – v/v/v). To remove white solids, the reaction mixture was filtered through a filter paper on a Büchner funnel with aspirator suction. The filtrate diluted with tap water (100 mL) and the mixture was extracted with ethyl acetate (100 mL × 1, 50 mL × 2). The combined organic layer was washed with brine (× 2) and filtered through a Celite pad on a Büchner funnel with aspirator suction, and dried over anhydrous Na_2SO_4. The solution was concentrated to dryness using a rotary evaporator and further dried *in vacuo*. The residue was purified by column chromatography (silica gel 96 g; hexane-ethyl acetate, 2:1 – v/v). The

obtained crude ester **3** was further purified by column chromatography (silica gel 40 g; chloroform-methanol, 98:2 – v/v) to give ester **3** (637 mg, 31% in 2 steps) as an orange-yellow powder. ^{1}H NMR (400 MHz, CD_3OD): δ 7.94 (d, *J* = 8.8 Hz, 1H), 7.36 (d, *J* = 2.4 Hz, 1H), 7.11 (dd, *J* = 2.4, 8.8 Hz, 1H), 4.47 (q, *J* = 7.2 Hz, 2H), 1.43 (t, *J* = 7.2 Hz, 3H); ^{13}C NMR (100 MHz, CD_3OD): δ 161.6, 159.4, 156.1, 147.8, 139.8, 126.5, 118.9, 107.2, 63.8, 14.5; HRMS (*m/z*): $[M+H]^+$ calcd. for $C_{10}H_{10}NO_3S$, 224.03759; found, 224.03736.

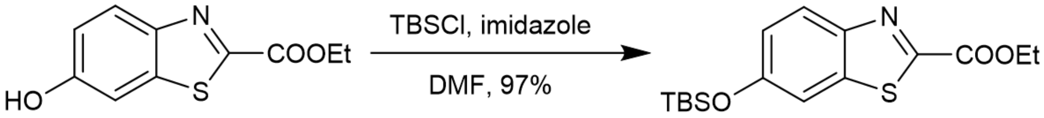

3 **4**

Preparation of TBS ether 4 from ester 3: Imidazole (697 mg, 10.2 mmol) and TBSCl (703 mg, 4.7 mmol) were added to a stirred solution of ester **3** (916 mg, 4.1 mmol) in DMF (13 mL). The mixture was stirred for 3 h at room temperature under a nitrogen atmosphere with monitoring by using TLC (hexane-ethyl acetate, 2:1 – v/v). The reaction mixture was quenched with a saturated aqueous NH_4Cl solution (45 mL). The mixture was extracted with ethyl acetate (50 mL × 3). The combined organic layer was washed with tap water (× 2) and brine (× 2), and dried over anhydrous Na_2SO_4. The solution was concentrated to dryness using a rotary evaporator and further dried *in vacuo*. The residue was purified by column chromatography (silica gel 69 g; hexane-ethyl acetate, 10:1 – v/v) to give TBS ether **4** (1.35 g, 97%) as a light yellow oil. ^{1}H NMR (400 MHz, $CDCl_3$): δ 8.07 (d, *J* = 9.2 Hz, 1H), 7.33 (d, *J* = 2.4 Hz, 1H), 7.06 (dd, *J* = 2.4, 8.8 Hz, 1H), 4.51 (q, *J* = 6.8 Hz, 2H), 1.45 (t, *J* = 7.2 Hz, 3H), 0.99 (s, 9H), 0.23 (s, 6H); ^{13}C NMR (100 MHz, $CDCl_3$): δ 160.0, 155.6, 155.1, 147.7, 137.8, 125.5, 120.9, 110.8, 62.2, 25.0, 17.6, 13.8, -5.0; HRMS (*m/z*): $[M+H]^+$ calcd. for $C_{16}H_{24}NO_3SiS$, 338.12407; found, 338.12405.

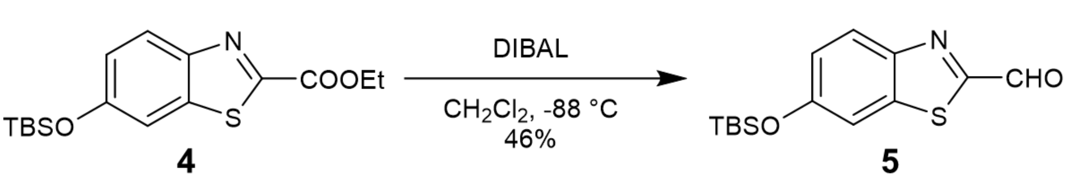

4 **5**

Preparation of aldehyde 5 from TBS ether 4: DIBAL (0.65 mL, 0.98 mmol, 1.5 M solution in toluene) was added dropwise in about 12 min to a stirred and cooled (-88 °C) solution of TBS ether **4** (303 mg, 0.90 mmol) in dry CH_2Cl_2 (15 mL) in an ultra-cooling reactor (model UCR-150N; Techno Sigma) cooled at -88 °C under a nitrogen atmosphere. The mixture was stirred at same temperature for 100 min under a nitrogen atmosphere with monitoring by using TLC (hexane-ethyl acetate, 2:1 – v/v).

The reaction mixture was quenched with ice-cooled methanol (0.65 mL) and stirred for 3 min. To the quenched solution was added a saturated aqueous potassium sodium tartrate solution (15 mL). The mixture was warmed to room temperature and stirred for 140 min. The resultant suspension was extracted with CH_2Cl_2 (15 mL × 3). The combined organic layer was washed with brine (70 mL × 1) and dried over anhydrous Na_2SO_4. The solution was concentrated to dryness using a rotary evaporator and further dried *in vacuo*. The residue was purified by column chromatography (silica gel 13 g; hexane-ethyl acetate, 20:1 – v/v) to give aldehyde **5** (122 mg, 46%) as a light yellow oil. ^1H NMR (400 MHz, $CDCl_3$): δ 10.09 (s, 1H), 8.07 (d, *J* = 9.2 Hz, 1H), 7.36 (d, *J* = 2.4 Hz, 1H), 7.11 (dd, *J* = 2.4, 9.2 Hz, 1H), 1.00 (s, 9H), 0.25 (s, 6H); ^{13}C NMR (100 MHz, $CDCl_3$): δ 185.1, 163.3, 156.6, 148.6, 138.2, 126.5, 122.1, 111.8, 25.5, 18.2, -4.4; HRMS (*m/z*): $[M+H]^+$ calcd. for $C_{14}H_{20}NO_2SiS$, 294.09785; found, 294.09748.

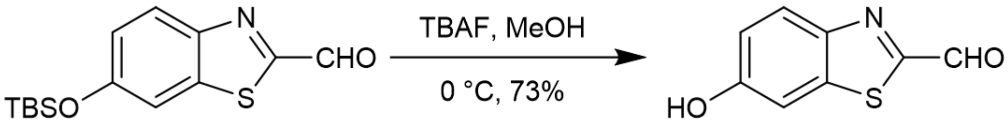

5 2

Preparation of compound 2 from aldehyde 5: TBAF (0.9 mL, 0.9 mmol, 1.0 M solution in THF) was added to a stirred and cooled (0 °C) solution of aldehyde **5** (229 mg, 0.78 mmol) in methanol (8 mL) in an ice bath under a nitrogen atmosphere. The mixture was stirred at same temperature for 80 min with monitoring by using TLC (hexane-ethyl acetate, 2:1 – v/v). The reaction mixture was quenched with a saturated aqueous NH_4Cl solution (40 mL) and diluted with water (40 mL). The mixture was extracted with ethyl acetate (80 mL × 3). The combined organic layer was washed with brine (× 2) and dried over anhydrous Na_2SO_4. The solution was concentrated to dryness using a rotary evaporator and further dried *in vacuo*. The residue was purified by column chromatography (silica gel 8 g; hexane-ethyl acetate, 2:1 – v/v) to give compound **2** (101 mg, 73%) as a light greenish-yellow powder. ^1H NMR (400 MHz, Acetone-d_6): δ 10.06 (s, 1H), 8.10 (d, *J* = 8.8 Hz, 1H), 7.57 (d, *J* = 2.4 Hz, 1H), 7.24 (dd, *J* = 2.4, 9.2 Hz, 1H); ^{13}C NMR (100 MHz, Acetone-d_6): δ 186.2, 163.3, 159.4, 148.6, 139.2, 127.4, 118.9, 107.7; HRMS (*m/z*): $[M+H]^+$ calcd. for $C_8H_6NO_2S$, 180.01138; found, 180.01160.

Supplementary Figures

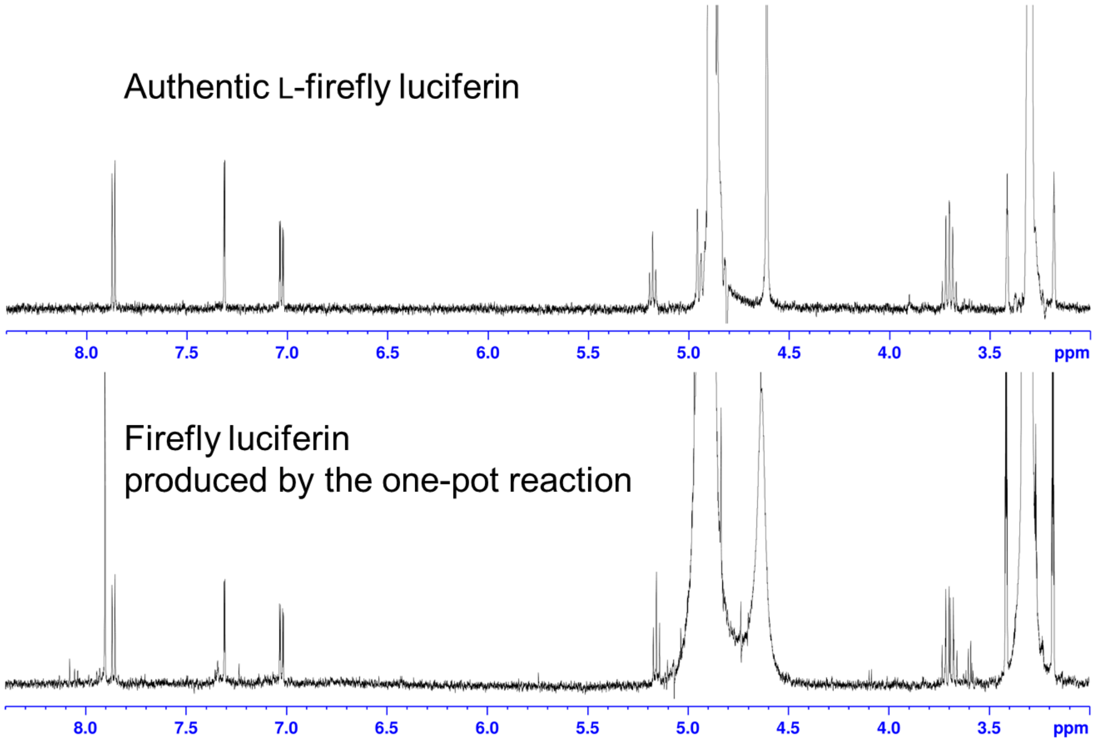

Supplementary Figure S1. ^{1}H NMR spectrum of firefly luciferin produced by the one-pot reaction of *p*-benzoquinone with L-cysteine in pH 7.5 Tris-HCl (600 MHz, CD_3OD). ^{1}H NMR data for firefly luciferin is shown in Supplementary Table S1.

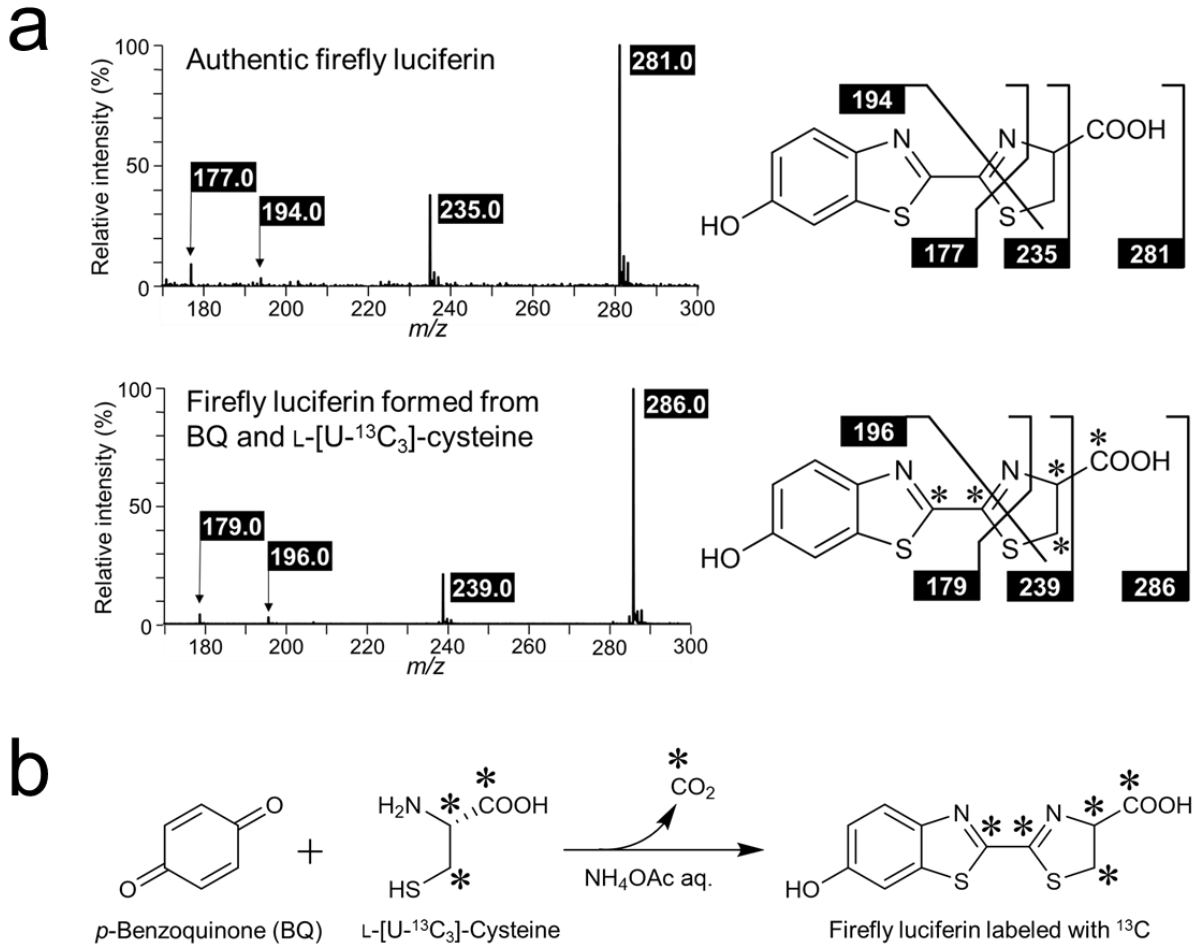

Supplementary Figure S2. MS analysis of firefly luciferin produced by the one-pot reaction of *p*-benzoquinone with L-[U-$^{13}C_3$]-cysteine. (a) Mass spectrum of firefly luciferin and predicted mass fragmentation. Asterisks indicate the position labeled with ^{13}C. (b) Reaction scheme of *p*-benzoquinone with L-[U-$^{13}C_3$]-cysteine to give L-firefly luciferin labeled with ^{13}C.

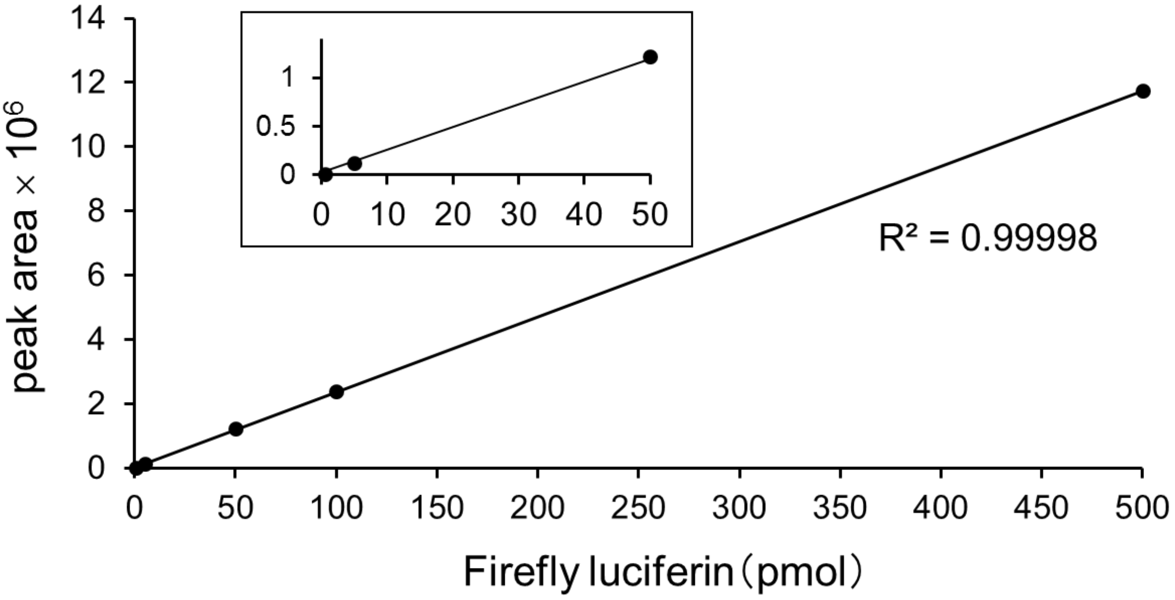

Supplementary Figure S3. Calibration curve of firefly luciferin determined by HPLC analysis with a fluorescence detector. Quantitative linearity (correlation efficient R^2 = 0.99998) was observed from 500 fmol to 500 pmol/injection. The box shows enlarged view of the range from 500 fmol to 50 pmol.

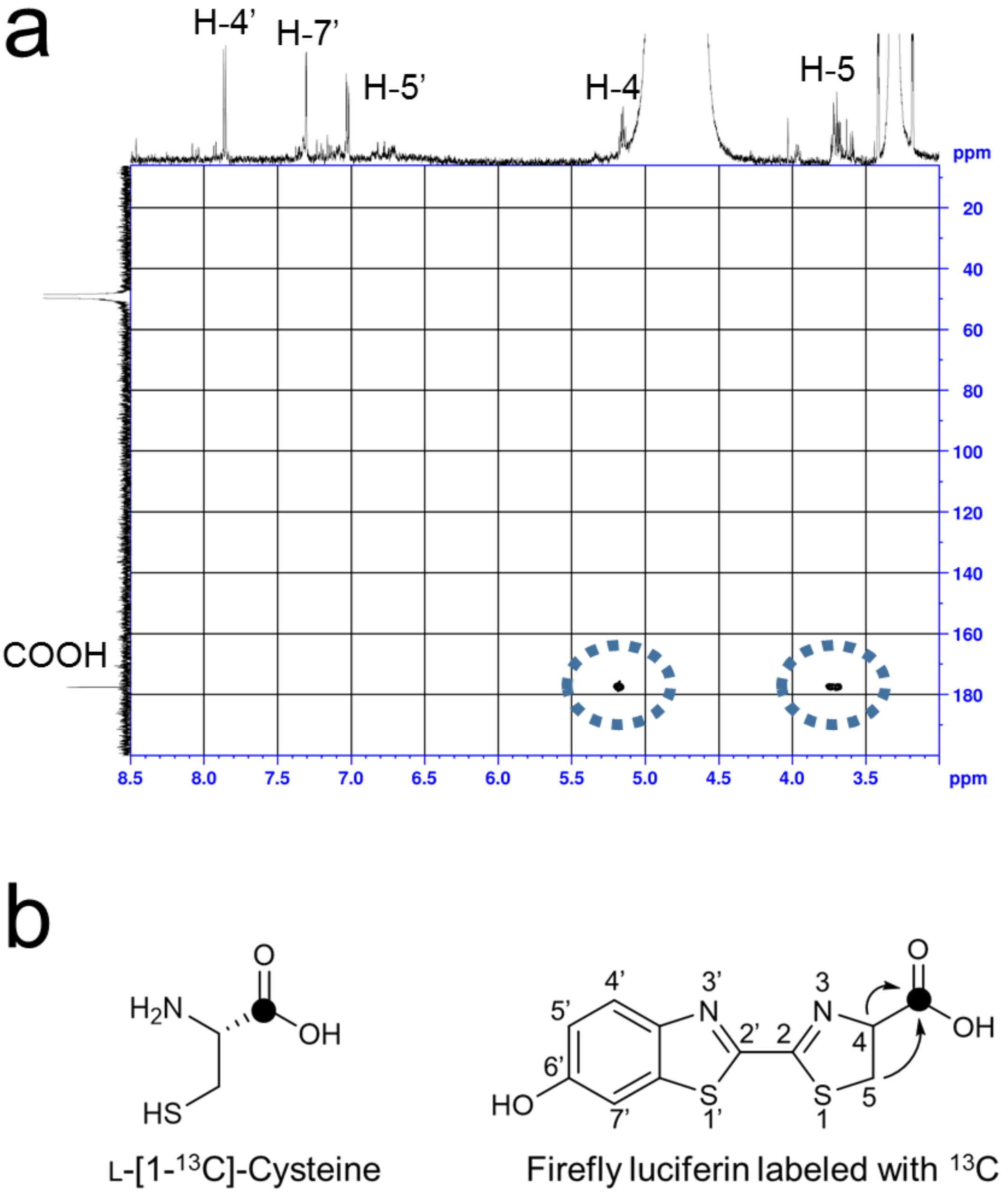

Supplementary Figure S4. HMBC analysis of firefly luciferin produced by the one-pot reaction of *p*-benzoquinone with L-[1-^{13}C]-cysteine. (a) HMBC spectrum of firefly luciferin produced by the reaction. (b) Chemical structures of L-[1-^{13}C]-cysteine and firefly luciferin produced by the reaction. Closed circle indicates the position labeled with ^{13}C. Arrows indicate HMBC correlations (from H to C).

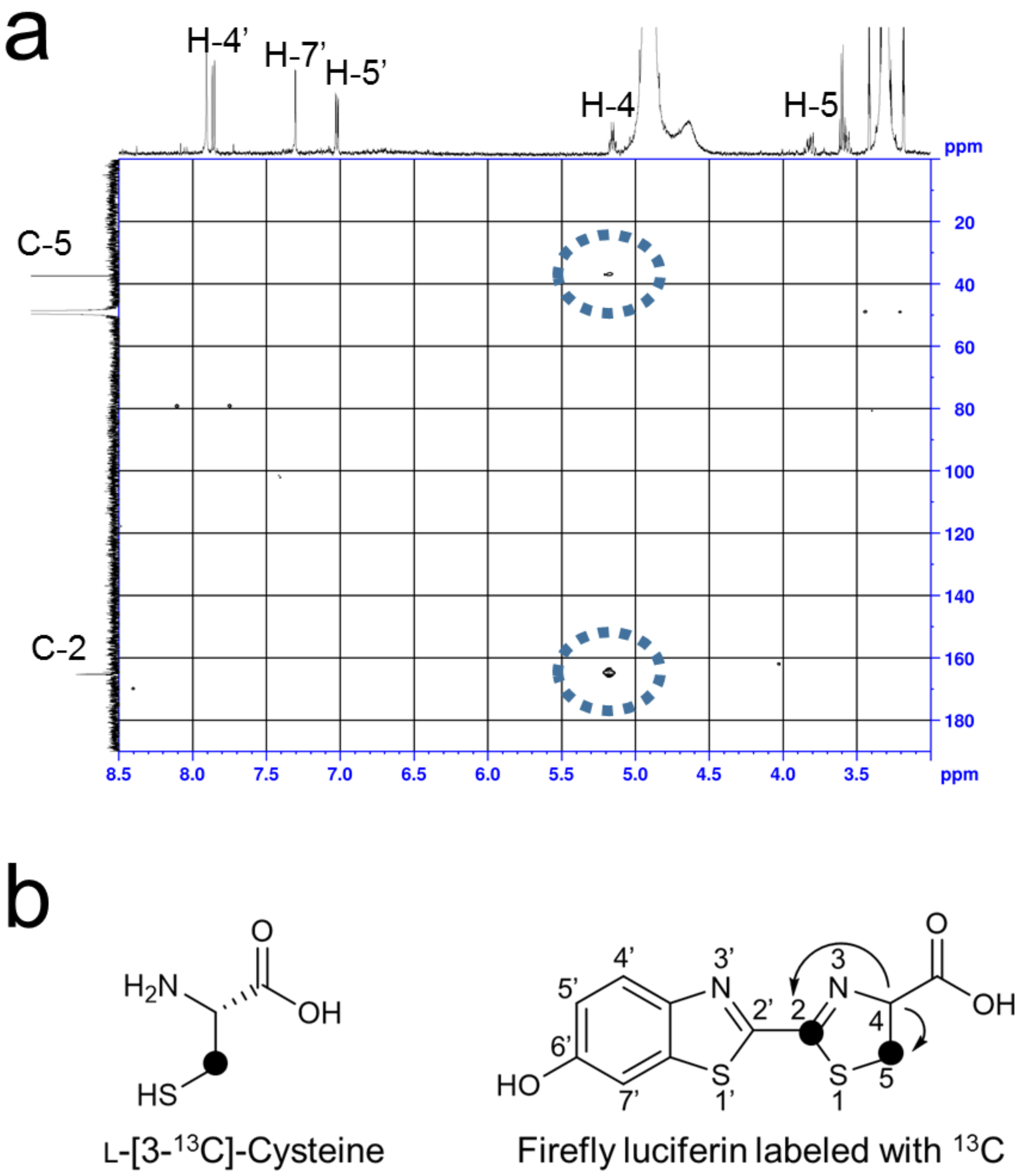

Supplementary Figure S5. HMBC analysis of firefly luciferin produced by the one-pot reaction of *p*-benzoquinone with L-[3-^{13}C]-cysteine. (a) HMBC spectrum of firefly luciferin produced by the reaction. (b) Chemical structures of L-[3-^{13}C]-cysteine and firefly luciferin produced by the reaction. Closed circle indicates the position labeled with ^{13}C. Arrows indicate HMBC correlations (from H to C).

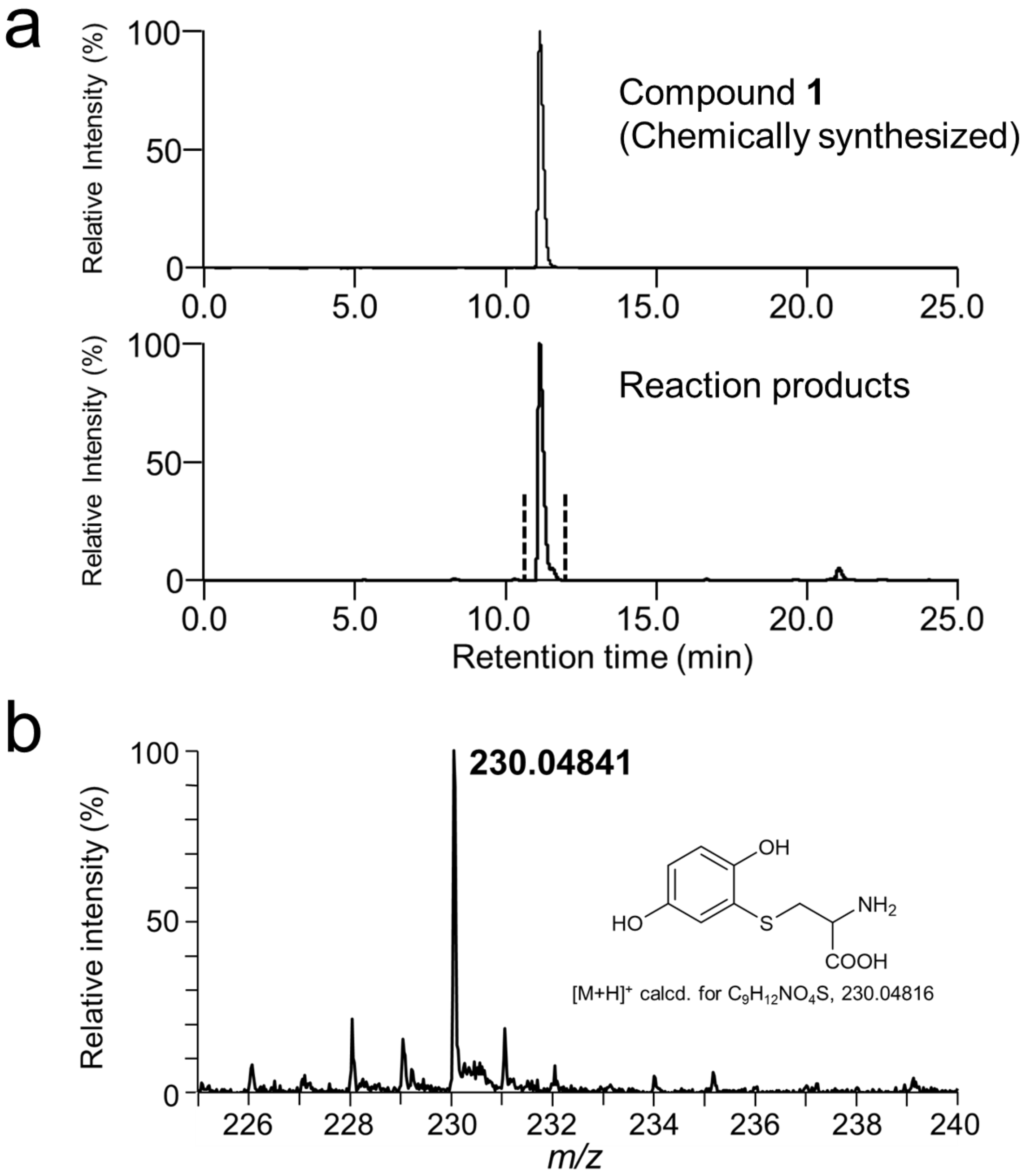

Supplementary Figure S6. Production of compound 1 by the one-pot reaction of *p*-benzoquinone with L-cysteine in a neutral buffer. (a) HPLC analysis of the reaction products by using a multiwavelength detector. The product between the vertical dashed lines was collected. (b) High resolution mass spectrum of the collected product.

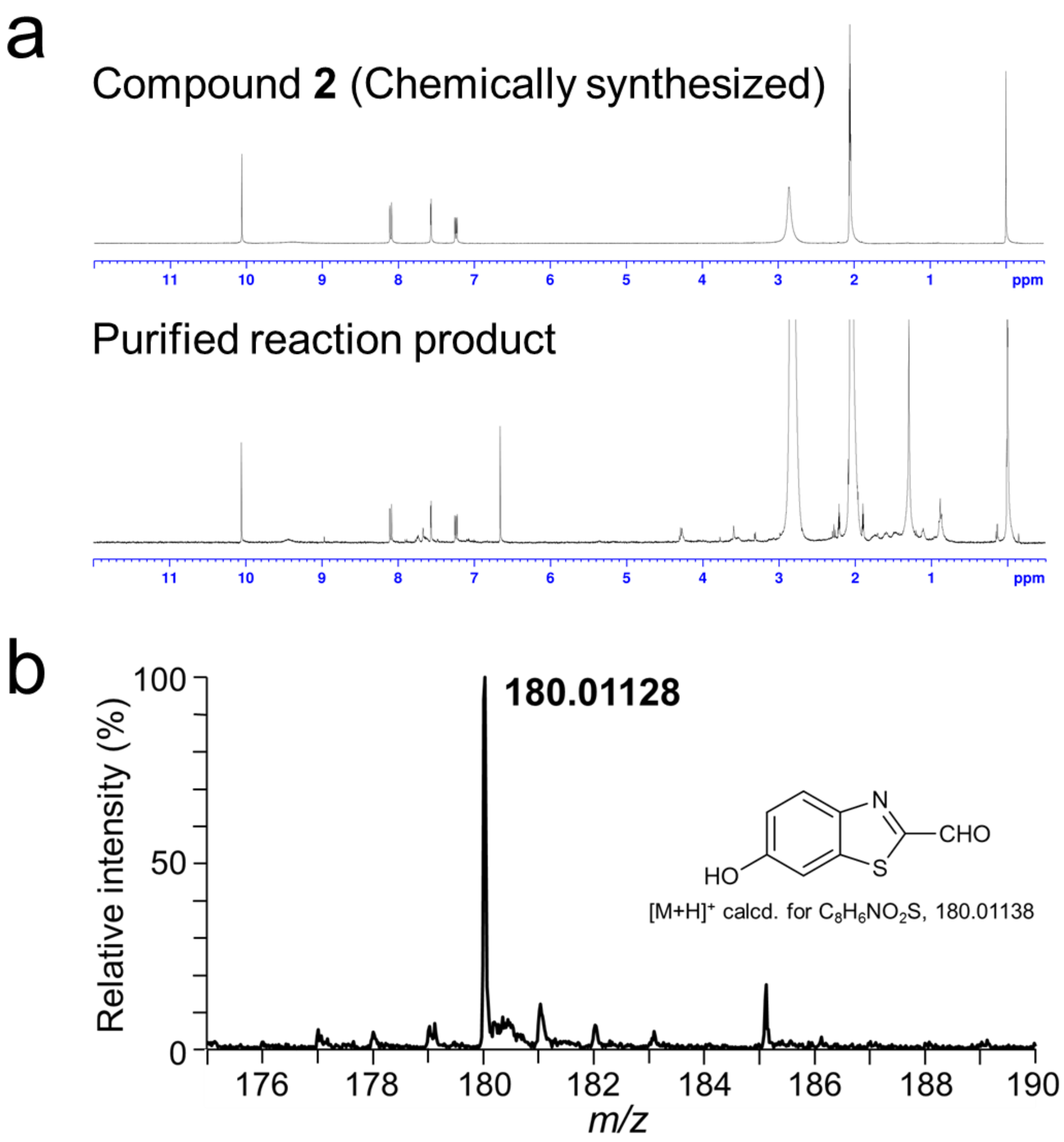

Supplementary Figure S7. Production of compound 2 by the one-pot reaction of *p*-benzoquinone with L-cysteine in a neutral buffer. (a) ^{1}H NMR spectra for chemically synthesized compound **2** (upper) and purified reaction product (lower) (400 MHz, acetone-d_6). (b) High resolution mass spectrum of the reaction product shown in panel a.

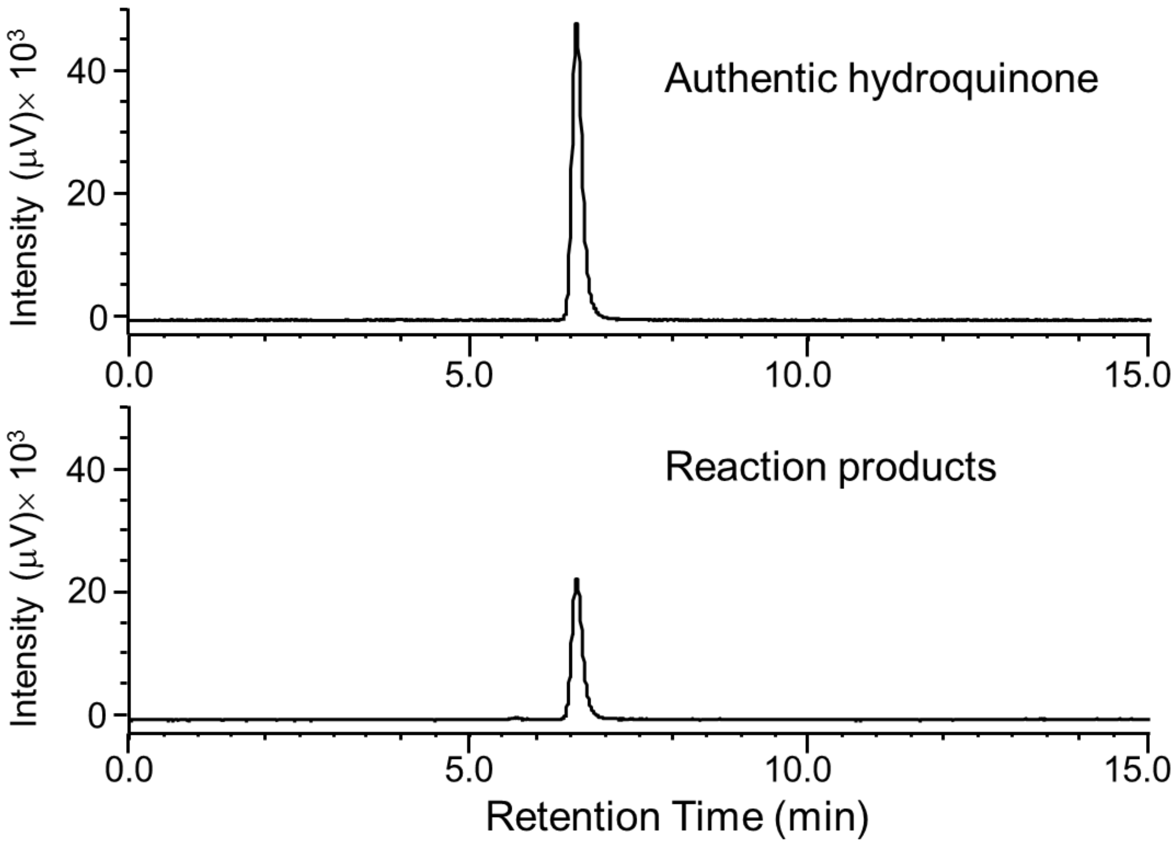

Supplementary Figure S8. Production of hydroquinone by the one-pot reaction of *p*-benzoquinone with L-cysteine in a neutral buffer. HPLC analysis of the reaction products by using a fluorescence detector. Upper panel, 200 pmol of authentic hydroquinone was subjected to HPLC analysis.

a

b

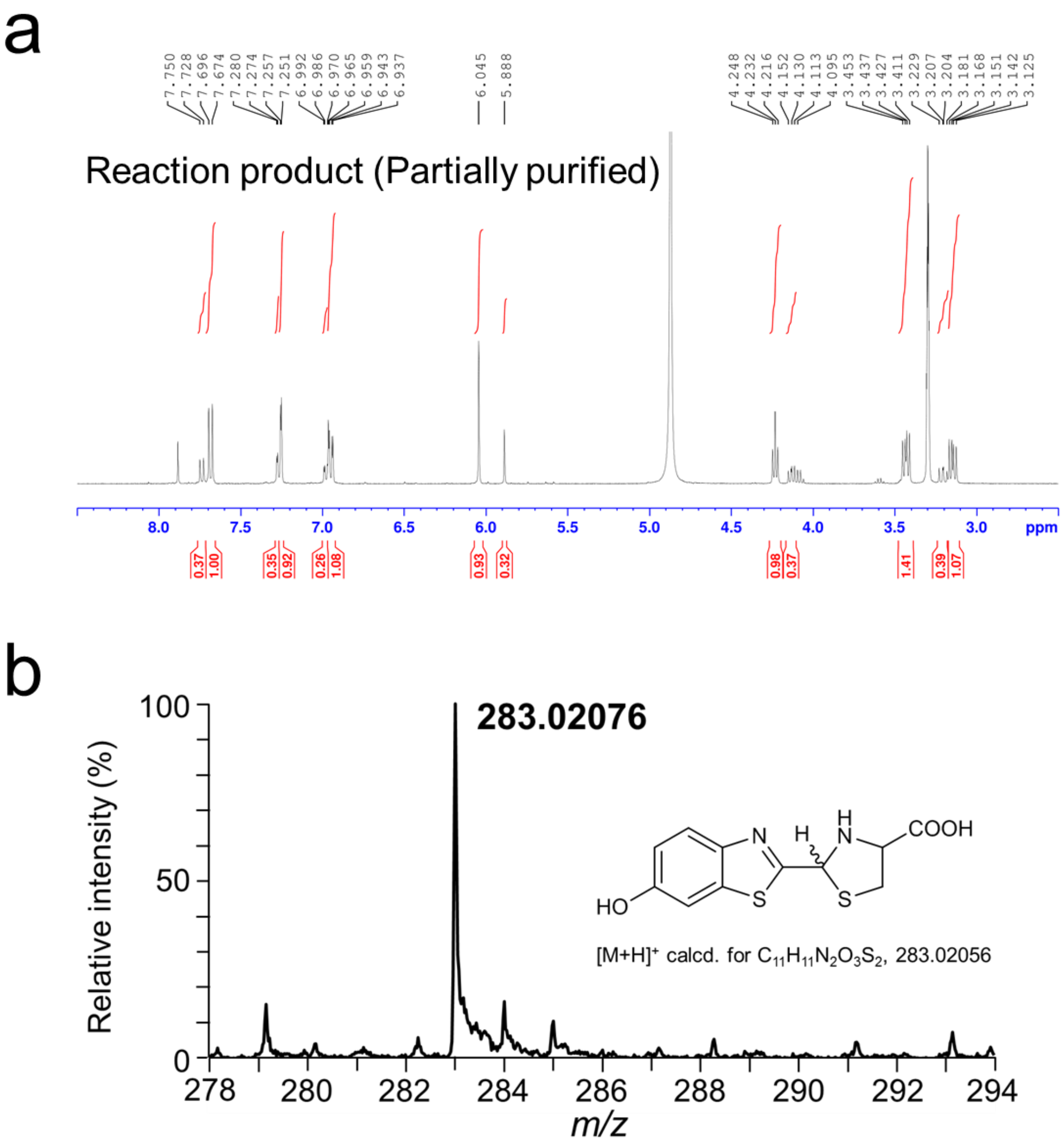

Supplementary Figure S9. Production of compound 3 by the reaction of compound 2 with D-cysteine in a neutral buffer. (a) ^{1}H NMR spectra for the ethyl acetate extract of the acidified resultant reaction mixture (400 MHz, CD_3OD). (b) High resolution mass spectrum of the reaction product shown in panel a.

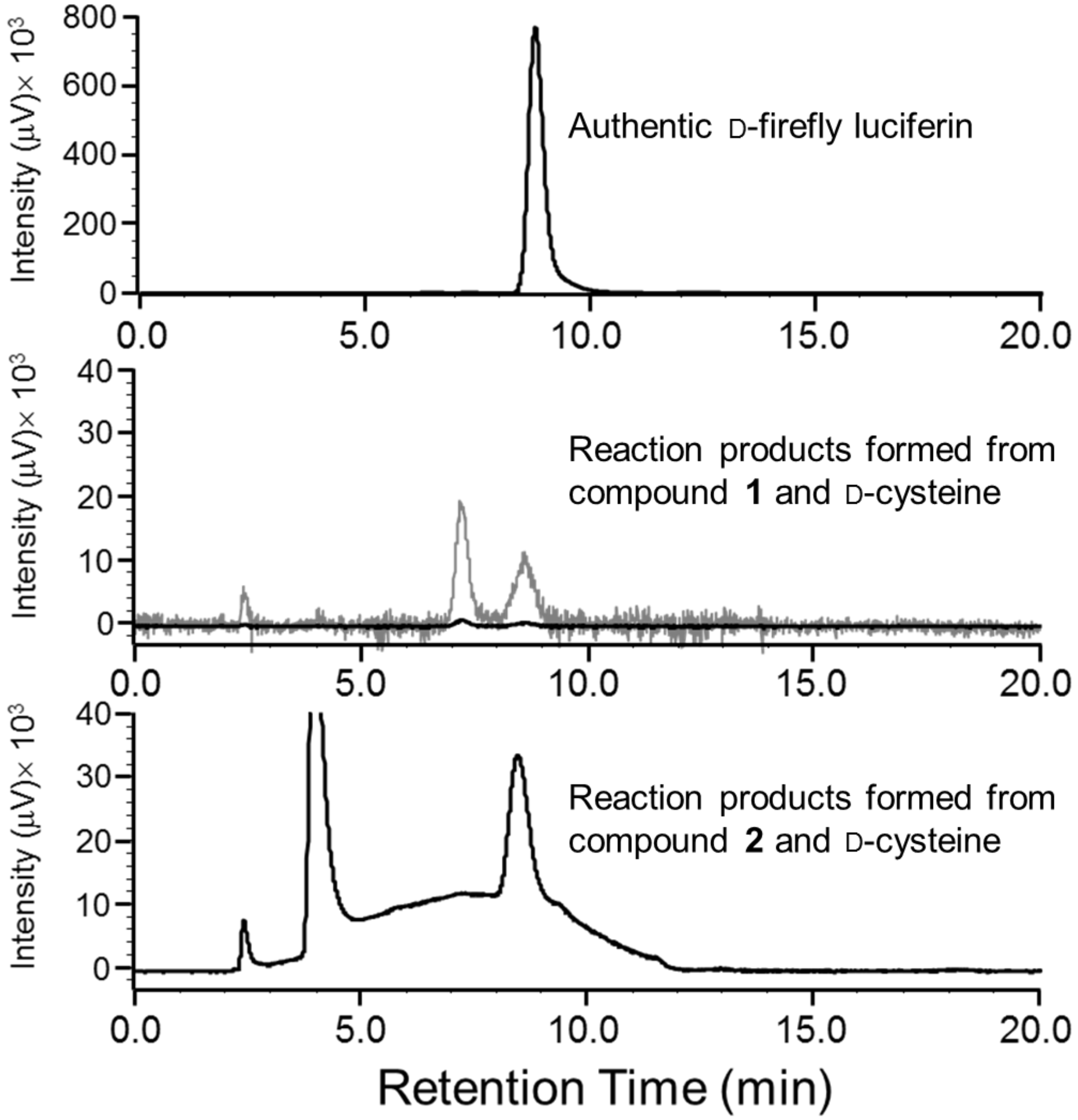

Supplementary Figure S10. HPLC analysis of reaction products formed from D-cysteine and compound 1 or 2 by using a fluorescence detector. Authentic D-firefly luciferin (500 pmol) was subjected to HPLC analysis. The gray line in the middle chromatogram shows vertically 40 times-enlarged view of the black line.

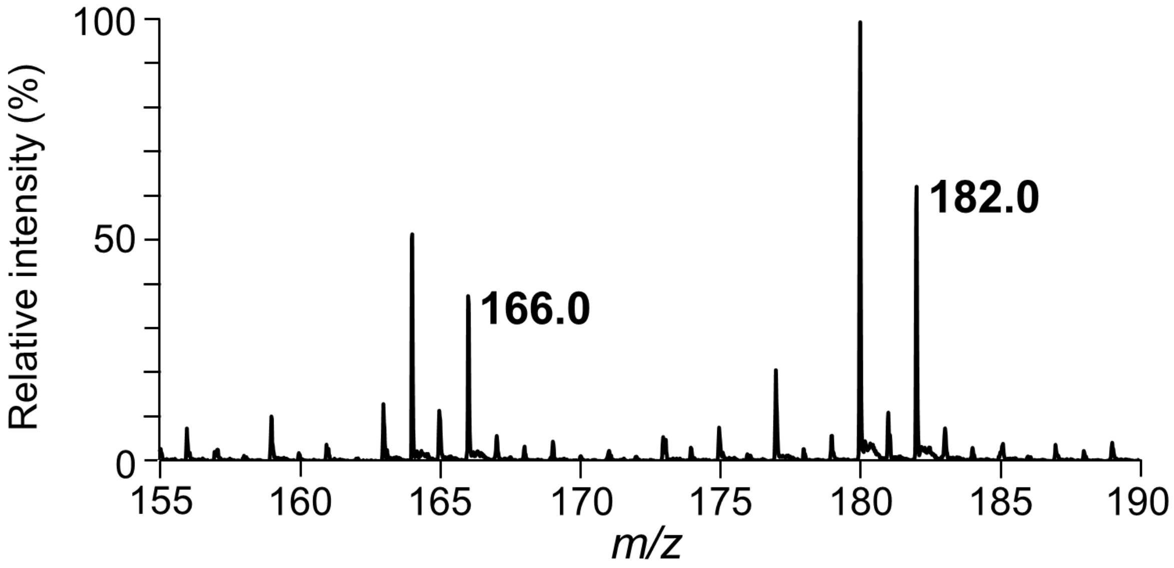

Supplementary Figure S11. Mass spectrum of the one-pot reaction products at a retention time of 4.2-5.1 min.

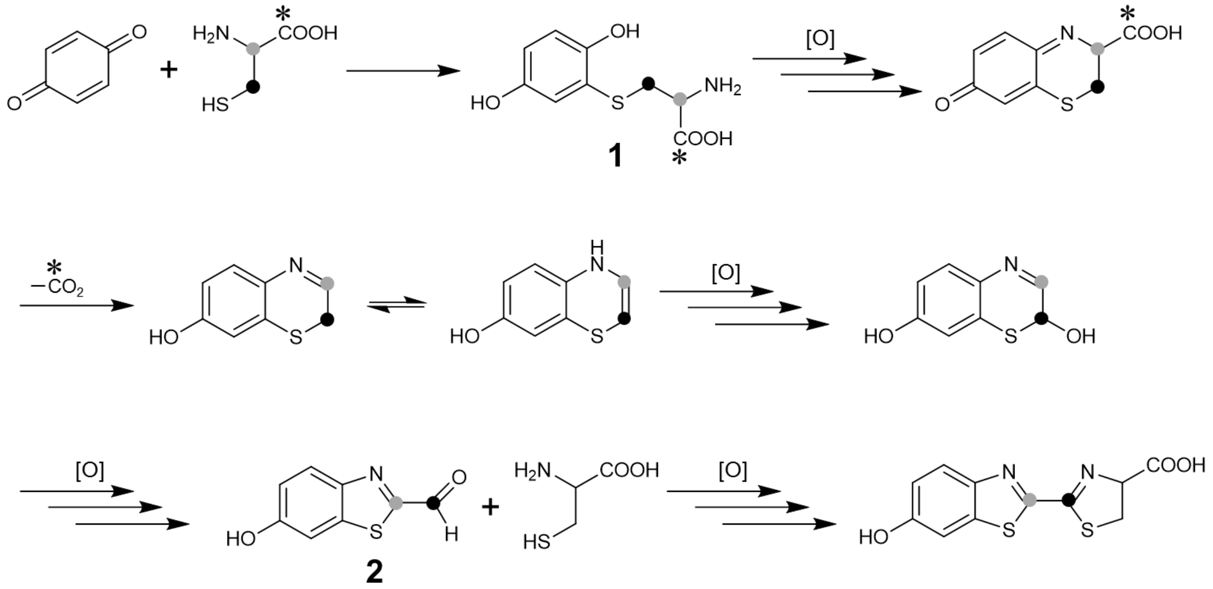

Supplementary Figure S12. Hypothetical reaction pathway for the one-pot formation of firefly luciferin *via* compound 1 and 2.

Supplementary tables

Supplementary Table S1. ^{1}H NMR data for firefly luciferin (600 MHz, CD_3OD).

	authentic L-firefly luciferin	firefly luciferin produced by the reaction
position	δ_H (multi, *J* in Hz)[a]	δ_H (multi, *J* in Hz)
5	3.68 (dd, 9.0, 10.8)	3.71 (dd, 9.2, 10.8)
	3.72 (dd, 9.0, 10.8)	3.75 (dd, 9.5, 10.8)
4	5.18 (t, 9.0)	5.19 (t, 9.3)
5’	7.03 (dd, 2.4, 9.0)	7.05 (dd, 2.4, 9.0)
7’	7.31 (d, 2.4)	7.33 (d, 2.4)
4’	7.87 (d, 9.0)	7.89 (d, 9.0)

[a] approximately 20 μg of authentic L-firefly luciferin was subjected to ^{1}H NMR analysis.

Supplementary Table S2. The yield of firefly luciferin in the one-pot reaction under various reaction conditions.

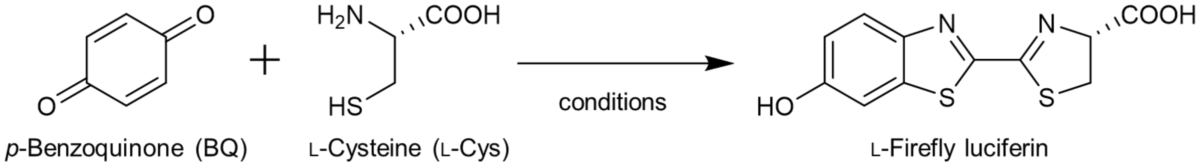

Entry	Solvent/buffer	BQ (mM)	L-Cys (mM)	BQ: L-Cys	Temp. (°C)	Yield (%)[a]
1	90 mM sodium citrate (pH 3.9)	4	4	1:1	30	ND
2	90 mM sodium acetate (pH 4.0)	4	4	1:1	30	trace
3	90 mM ammonium acetate (pH 7.0)	4	4	1:1	30	0.30 ± 0.24[b]
4	90 mM KH_2PO_4-KOH (pH 7.5)	4	4	1:1	30	0.35 ± 0.10[c]
5	90 mM HEPES-KOH (pH 7.5)	4	4	1:1	30	0.45 ± 0.06[c]
6	90 mM Tris-HCl (pH 7.5)	4	4	1:1	30	0.29 ± 0.12[c]
7	90 mM carbonate-bicarbonate (pH 9.5)	4	4	1:1	30	trace
8	90 mM glycine-NaOH (pH 9.5)	4	4	1:1	30	trace
9	water	4	4	1:1	30	trace
10	90% ethanol-water	4	4	1:1	30	trace
11	90% methanol-water	4	4	1:1	30	ND
12	90% acetonitrile-water	4	4	1:1	30	ND
13	45 mM ammonium acetate (pH 7.0)	4	4	1:1	30	0.39 ± 0.42
14	225 mM ammonium acetate (pH 7.0)	4	4	1:1	30	0.72 ± 0.59
15	450 mM ammonium acetate (pH 7.0)	4	4	1:1	30	0.20 ± 0.12
16	45 mM Tris-HCl (pH 7.5)	4	4	1:1	30	0.13 ± 0.03
17	225 mM Tris-HCl (pH 7.5)	4	4	1:1	30	0.10 ± 0.01
18	450 mM Tris-HCl (pH 7.5)	4	4	1:1	30	0.10 ± 0.05
19	90 mM ammonium acetate (pH 7.0)	0.8	4	1:5	30	0.24 ± 0.25[d]
20	90 mM ammonium acetate (pH 7.0)	0.8	1.6	1:2	30	0.37 ± 0.35
21	90 mM ammonium acetate (pH 7.0)	0.8	0.8	1:1	30	0.23 ± 0.23
22	90 mM ammonium acetate (pH 7.0)	1.6	0.8	2:1	30	ND
23	90 mM ammonium acetate (pH 7.0)	4	0.8	5:1	30	ND
24	90 mM Tris-HCl (pH 7.5)	0.8	4	1:5	30	0.20 ± 0.04[d]
25	90 mM Tris-HCl (pH 7.5)	0.8	1.6	1:2	30	0.15 ± 0.06
26	90 mM Tris-HCl (pH 7.5)	0.8	0.8	1:1	30	0.18 ± 0.13
27	90 mM Tris-HCl (pH 7.5)	1.6	0.8	2:1	30	ND
28	90 mM Tris-HCl (pH 7.5)	4	0.8	5:1	30	ND
29	90 mM universal buffer (pH 4.0)	4	4	1:1	30	ND
30	90 mM universal buffer (pH 5.0)	4	4	1:1	30	trace

31	90 mM universal buffer (pH 6.0)	4	4	1:1	30	0.14 ± 0.09[c]
32	90 mM universal buffer (pH 7.0)	4	4	1:1	30	0.26 ± 0.24[c]
33	90 mM universal buffer (pH 7.5)	4	4	1:1	30	0.13 ± 0.08[c]
34	90 mM universal buffer (pH 8.6)	4	4	1:1	30	trace
35	90 mM universal buffer (pH 9.5)	4	4	1:1	30	trace
36[e,f]	90 mM ammonium acetate (pH 7.0)	4	4	1:1	4	trace
37[e,f]	90 mM ammonium acetate (pH 7.0)	4	4	1:1	60	0.70 ± 0.26
38[e,f]	90 mM ammonium acetate (pH 7.0)	4	4	1:1	90	0.22 ± 0.18
39[f,g,h,i]	90 mM ammonium acetate (pH 7.0)	4	4	1:1	30	trace
40[f,g,h,j]	90 mM ammonium acetate (pH 7.0)	4	4	1:1	30	0.05 ± 0.04
41[k]	90 mM ammonium acetate (pH 7.0)	4	4	1:1	30	0.06 ± 0.03
42[l]	90 mM ammonium acetate (pH 7.0)	4	4	1:1	30	0.14 ± 0.07
43[m]	90 mM ammonium acetate (pH 7.0)	4	4	1:1	30	0.10 ± 0.02
44[n]	90 mM ammonium acetate (pH 7.0)	4	4	1:1	30	0.34 ± 0.18
45	200 mM ammonium acetate (pH 7.0)	40	40	1:1	30	0.13 ± 0.06
46	200 mM ammonium acetate (pH 7.0)	20	20	1:1	30	0.21 ± 0.11
47	200 mM ammonium acetate (pH 7.0)	10	10	1:1	30	0.18 ± 0.14
48	200 mM ammonium acetate (pH 7.0)	4	4	1:1	30	0.33 ± 0.29
49	200 mM ammonium acetate (pH 7.0)	0.8	0.8	1:1	30	0.19 ± 0.23
50[o]	90 mM ammonium acetate (pH 7.0)	4	4	1:1	30	ND
51[o,p]	90 mM ammonium acetate (pH 7.0)	4	4	1:1	30	ND

Concentrations of *p*-benzoquinone and L-cysteine in this table indicate a final concentration in the reaction mixture. Reaction was carried out for 3 h under an air atmosphere unless otherwise noted. Each reaction was performed in triplicate unless otherwise noted. ND and trace indicate "not detected" and "the yield of firefly luciferin was less than 0.005%", respectively. [a] Yields based on cysteine were determined by HPLC analysis with calibration curve shown in Supplementary Figure S3. Mean ± SD for n = 3 unless otherwise noted. [b] Mean ± SD for n = 14. [c] Mean ± SD for n = 4.[d] Yield was based on *p*-benzoquinone. [e] Carried out in a test tube. [f] With stirring by using a magnetic stirrer. [g] Carried out in a eggplant flask. [h] Solutions of *p*-benzoquinone and cysteine were degassed by freeze-pump-thaw. [i] Under a nitrogen gas. [j] Under a 95% oxygen gas. [k] For 30 min. [l] For 60 min. [m] For 90 min. [n] For 120 min. [o] 1,4-hydoquinone was used instead of *p*-benzoquinone. [p] For 360 min.

Supplementary References

1. White, E. H., McCapra, F., Field, G. F. & McElroy, W. D. The structure and synthesis of firefly luciferin. *J. Am. Chem. Soc.* **83,** 2402-2403 (1961).
2. Hayashi, H. & Koshimizu, K. Methoxy-1,4-benzoquinone and methyl gallate, inhibitory factors of betacyanin synthesis in *Amaranthus*, from persimmon fruits. *Agric. Biol. Chem.* **43,** 113-116 (1979).
3. Kociok-Köhn, G. & Lewis, S. E. Crystallographic rationalization of the reactivity and spectroscopic properties of (2*R*)-*S*-(2,5-dihydroxyphenyl)cysteine. *Acta Cryst.* **C66,** 187-189 (2010).
4. Löwik, D. W. P. M., Tisi, L. C., Murray, J. A. H. & Lowe, C. R. Synthesis of 6-hydroxybenzothiazole-2-carboxylic acid. *Synthesis* **12,** 1780-1783 (2001).
5. Meroni, G., Rajabi, M., Ciana, P., Maggi, A. & Santaniello, E. Synthesis of 2-substituted-6-hydroxy and 6-methoxy benzothiazoles from 1,4-benzoquinone. *ARKIVOC* **6,** 53-60 (2010).
6. Ciuffreda, P., Casati, S., Meroni, G & Santaniello. A new synthesis of dehydroluciferin [2-(6’-hydroxy-2’-benzothiazolyl)-thiazole-4-carboxylic acid] from 1,4-benzoquinone. *Tetrahedron* **69,** 5893-5897 (2013).

Spectra of synthesized compounds

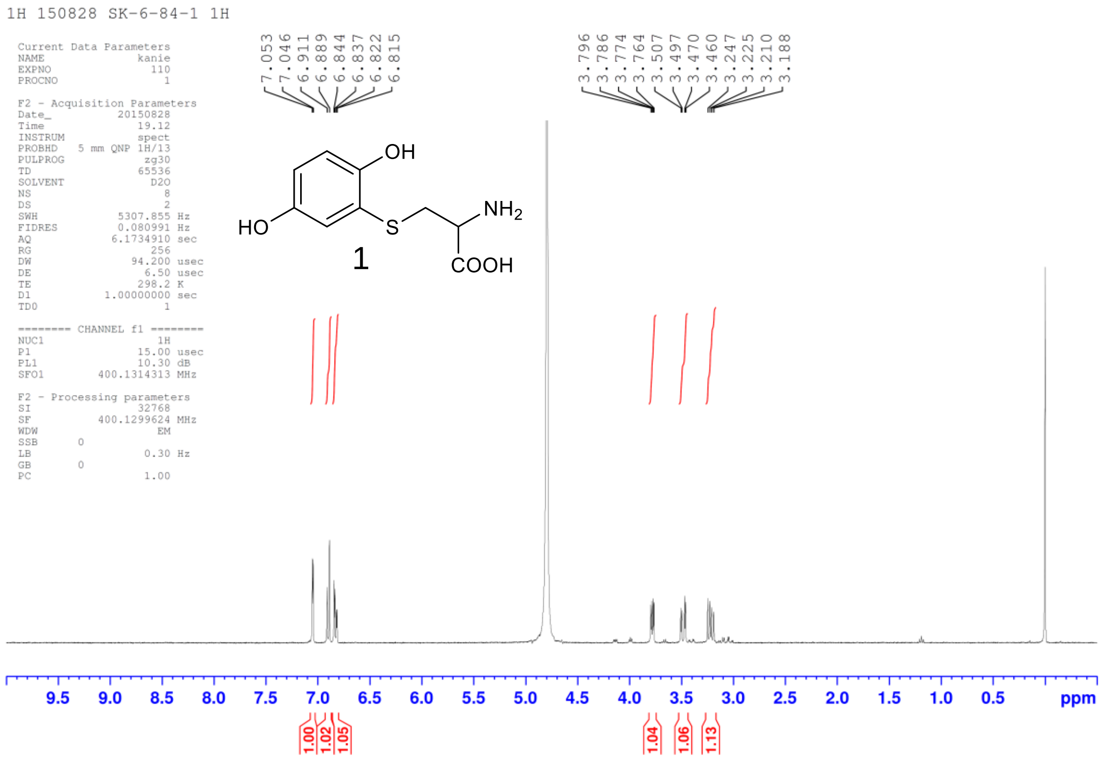

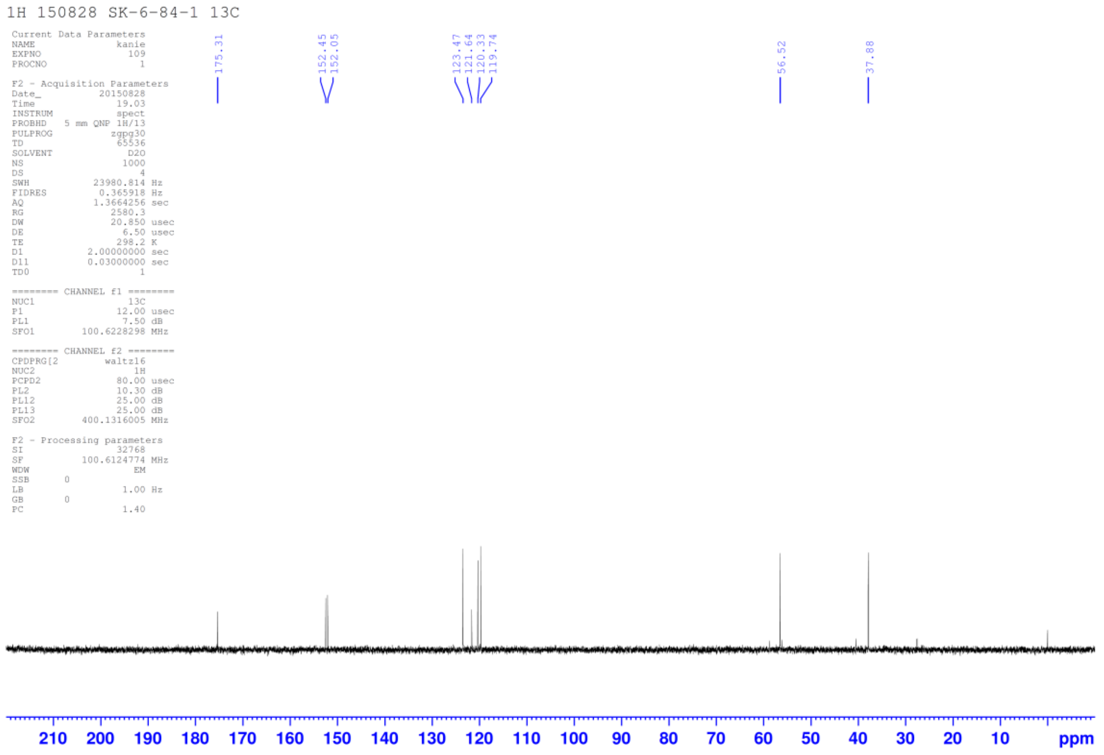

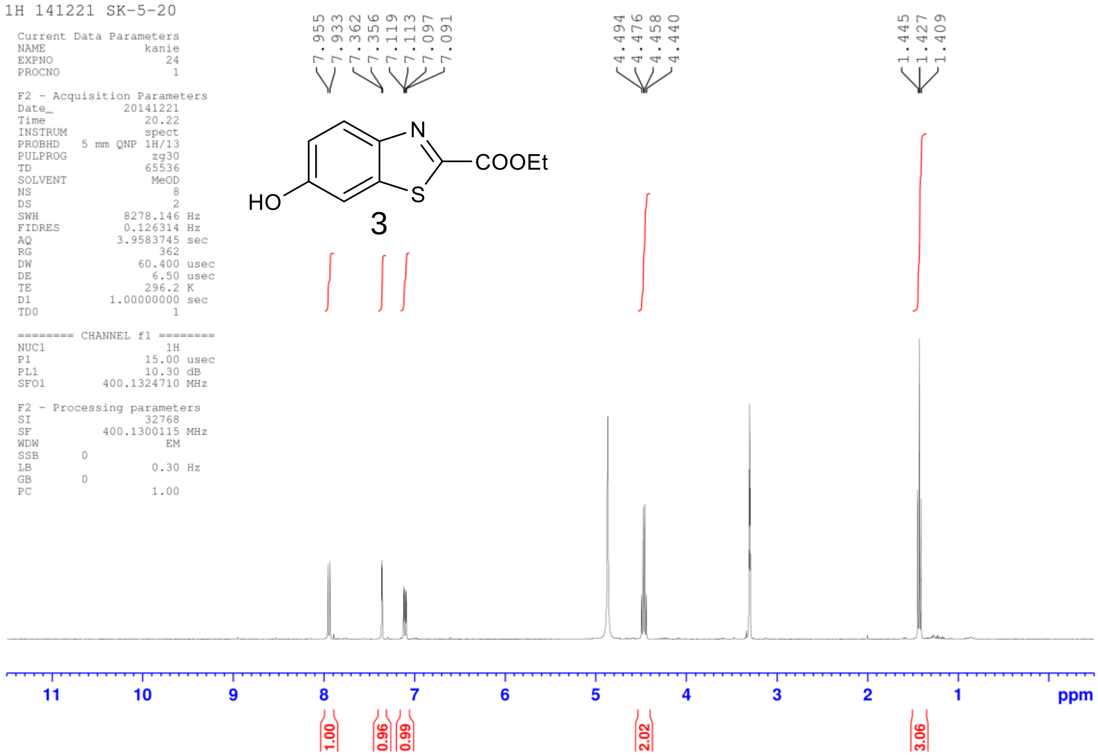
1H 141221 SK-5-20
Current Data Parameters
NAME kanie
EXPNO 24
PROCNO 1
F2 - Acquisition Parameters
Date_ 20141221
Time 20.22
INSTRUM spect
PROBHD 5 mm QNP 1H/13
PULPROG zg30
TD 65536
SOLVENT MeOD
NS 8
DS 2
SWH 8278.146 Hz
FIDRES 0.126314 Hz
AQ 3.9583745 sec
RG 362
DW 60.400 usec
DE 6.50 usec
TE 296.2 K
D1 1.00000000 sec
TD0 1
======== CHANNEL f1 ========
NUC1 1H
P1 15.00 usec
PL1 10.30 dB
SFO1 400.1324710 MHz
F2 - Processing parameters
SI 32768
SF 400.1300115 MHz
WDW EM
SSB 0
LB 0.30 Hz
GB 0
PC 1.00
7.955
7.933
7.362
7.356
7.119
7.113
7.097
7.091
4.494
4.476
4.458
4.440
1.445
1.427
1.409
HO
N
S
COOEt
3
11 10 9 8 7 6 5 4 3 2 1 ppm
1.00
0.96
0.99
2.02
3.06

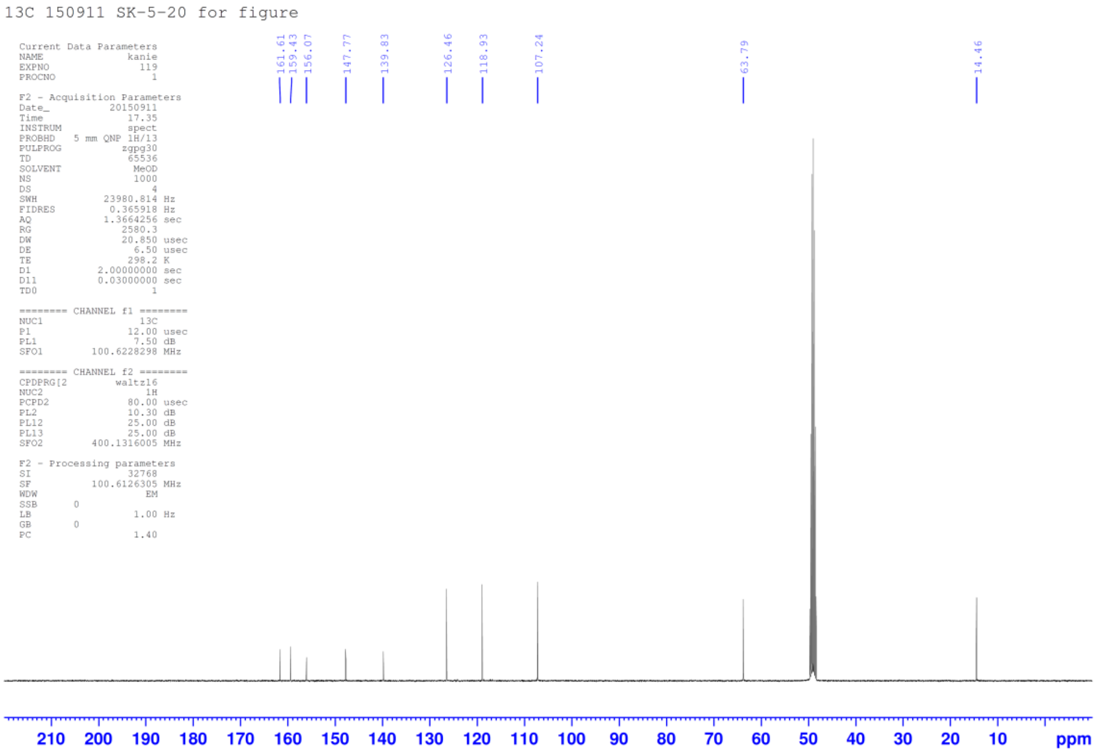
13C 150911 SK-5-20 for figure
Current Data Parameters
NAME kanie
EXPNO 119
PROCNO 1
F2 - Acquisition Parameters
Date_ 20150911
Time 17.35
INSTRUM spect
PROBHD 5 mm QNP 1H/13
PULPROG zgpg30
TD 65536
SOLVENT MeOD
NS 1000
DS 4
SWH 23980.814 Hz
FIDRES 0.365918 Hz
AQ 1.3664256 sec
RG 2580.3
DW 20.850 usec
DE 6.50 usec
TE 298.2 K
D1 2.00000000 sec
D11 0.03000000 sec
TD0 1
======== CHANNEL f1 ========
NUC1 13C
P1 12.00 usec
PL1 7.50 dB
SFO1 100.6228298 MHz
======== CHANNEL f2 ========
CPDPRG[2 waltz16
NUC2 1H
PCPD2 80.00 usec
PL2 10.30 dB
PL12 25.00 dB
PL13 25.00 dB
SFO2 400.1316005 MHz
F2 - Processing parameters
SI 32768
SF 100.6126305 MHz
WDW EM
SSB 0
LB 1.00 Hz
GB 0
PC 1.40
161.61
159.43
156.07
147.77
139.83
126.46
118.93
107.24
63.79
14.46
210 200 190 180 170 160 150 140 130 120 110 100 90 80 70 60 50 40 30 20 10 ppm

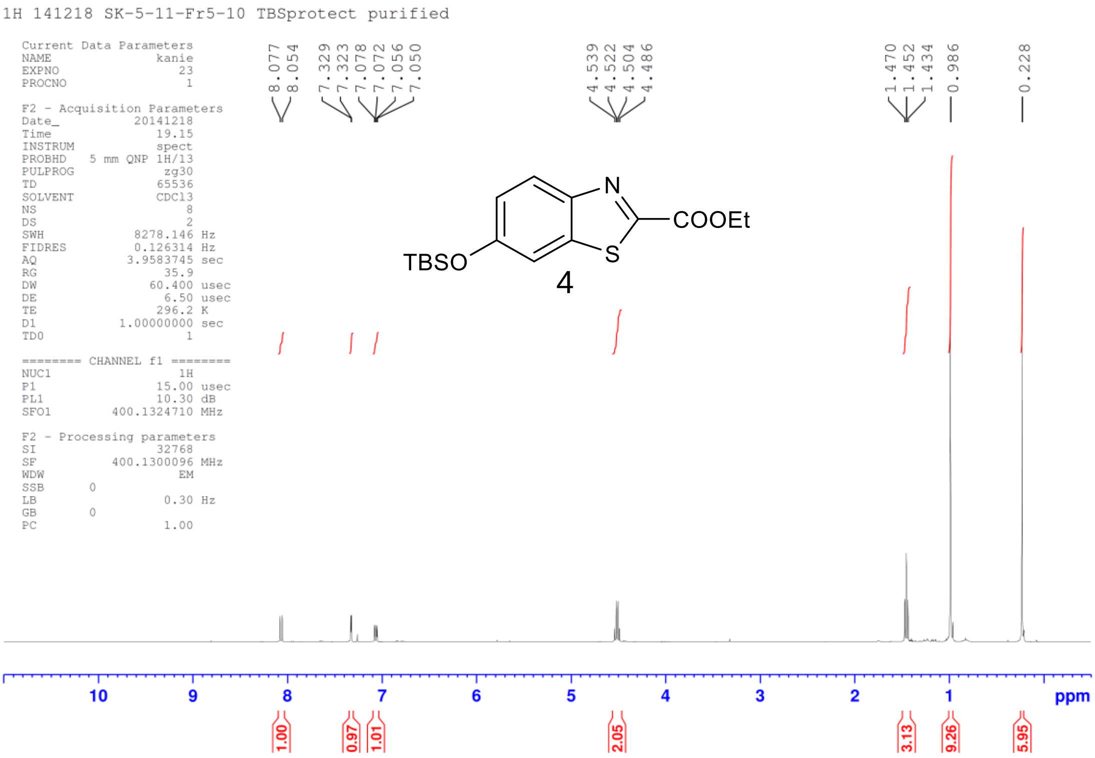
1H 141218 SK-5-11-Fr5-10 TBSprotect purified
Current Data Parameters
NAME kanie
EXPNO 23
PROCNO 1
F2 - Acquisition Parameters
Date_ 20141218
Time 19.15
INSTRUM spect
PROBHD 5 mm QNP 1H/13
PULPROG zg30
TD 65536
SOLVENT CDCl3
NS 8
DS 2
SWH 8278.146 Hz
FIDRES 0.126314 Hz
AQ 3.9583745 sec
RG 35.9
DW 60.400 usec
DE 6.50 usec
TE 296.2 K
D1 1.00000000 sec
TD0 1
======== CHANNEL f1 ========
NUC1 1H
P1 15.00 usec
PL1 10.30 dB
SFO1 400.1324710 MHz
F2 - Processing parameters
SI 32768
SF 400.1300096 MHz
WDW EM
SSB 0
LB 0.30 Hz
GB 0
PC 1.00
8.077
8.054
7.329
7.323
7.078
7.072
7.056
7.050
4.539
4.522
4.504
4.486
1.470
1.452
1.434
0.986
0.228
N
S
COOEt
TBSO
4
1.00
0.97
1.01
2.05
3.13
9.26
5.95
10
9
8
7
6
5
4
3
2
1
ppm

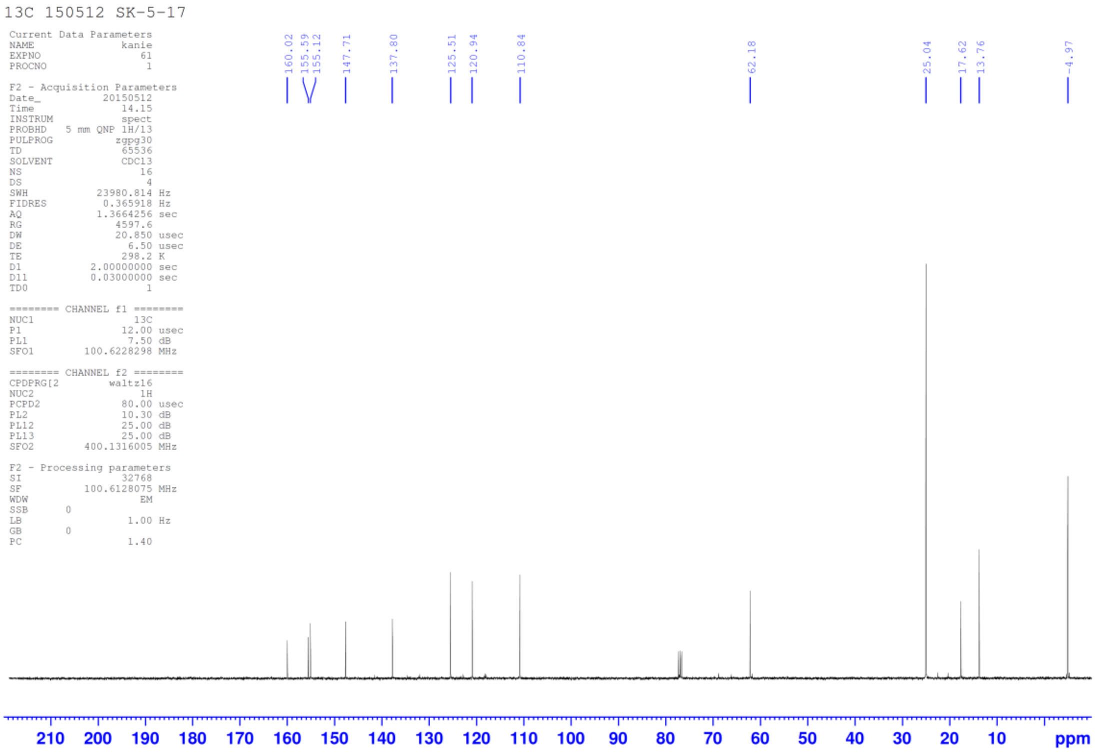
13C 150512 SK-5-17
Current Data Parameters
NAME kanie
EXPNO 61
PROCNO 1
F2 - Acquisition Parameters
Date_ 20150512
Time 14.15
INSTRUM spect
PROBHD 5 mm QNP 1H/13
PULPROG zgpg30
TD 65536
SOLVENT CDCl3
NS 16
DS 4
SWH 23980.814 Hz
FIDRES 0.365918 Hz
AQ 1.3664256 sec
RG 4597.6
DW 20.850 usec
DE 6.50 usec
TE 298.2 K
D1 2.00000000 sec
D11 0.03000000 sec
TD0 1
======== CHANNEL f1 ========
NUC1 13C
P1 12.00 usec
PL1 7.50 dB
SFO1 100.6228298 MHz
======== CHANNEL f2 ========
CPDPRG[2 waltz16
NUC2 1H
PCPD2 80.00 usec
PL2 10.30 dB
PL12 25.00 dB
PL13 25.00 dB
SFO2 400.1316005 MHz
F2 - Processing parameters
SI 32768
SF 100.6128075 MHz
WDW EM
SSB 0
LB 1.00 Hz
GB 0
PC 1.40
160.02
155.59
155.12
147.71
137.80
125.51
120.94
110.84
62.18
25.04
17.62
13.76
-4.97
210
200
190
180
170
160
150
140
130
120
110
100
90
80
70
60
50
40
30
20
10
ppm

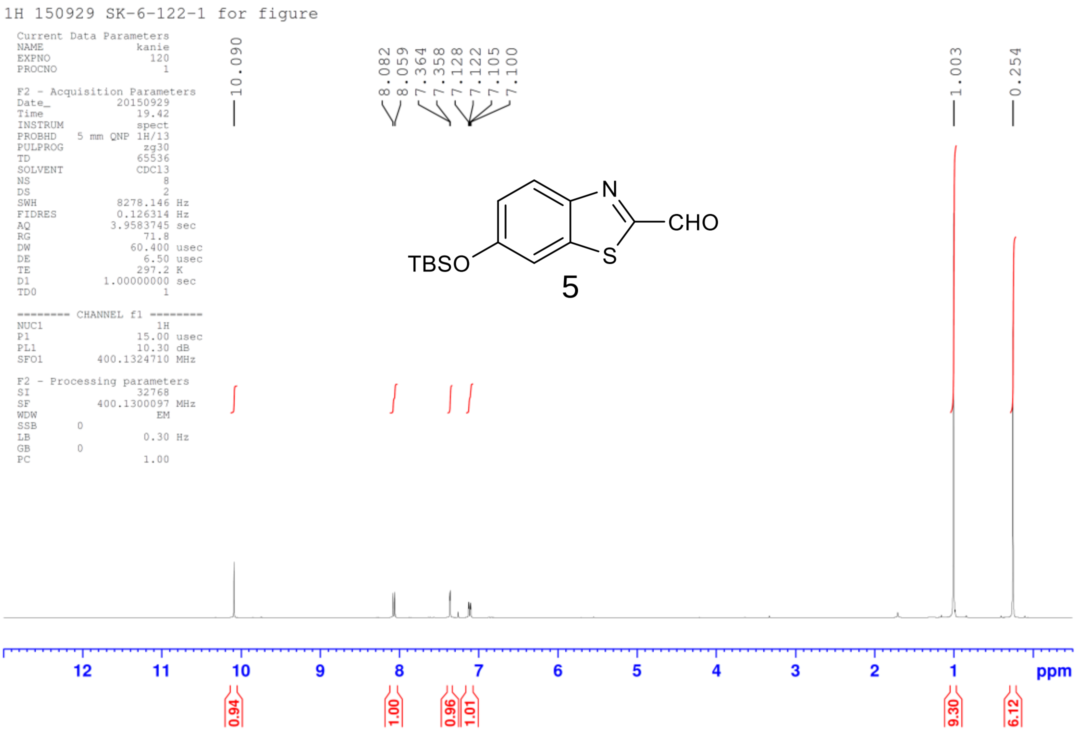
1H 150929 SK-6-122-1 for figure
Current Data Parameters
NAME kanie
EXPNO 120
PROCNO 1
F2 - Acquisition Parameters
Date_ 20150929
Time 19.42
INSTRUM spect
PROBHD 5 mm QNP 1H/13
PULPROG zg30
TD 65536
SOLVENT CDCl3
NS 8
DS 2
SWH 8278.146 Hz
FIDRES 0.126314 Hz
AQ 3.9583745 sec
RG 71.8
DW 60.400 usec
DE 6.50 usec
TE 297.2 K
D1 1.00000000 sec
TD0 1
======== CHANNEL f1 ========
NUC1 1H
P1 15.00 usec
PL1 10.30 dB
SFO1 400.1324710 MHz
F2 - Processing parameters
SI 32768
SF 400.1300097 MHz
WDW EM
SSB 0
LB 0.30 Hz
GB 0
PC 1.00
10.090
8.082
8.059
7.364
7.358
7.128
7.122
7.105
7.100
1.003
0.254
N
S
CHO
TBSO
5
ppm
0.94
1.00
0.96
1.01
9.30
6.12

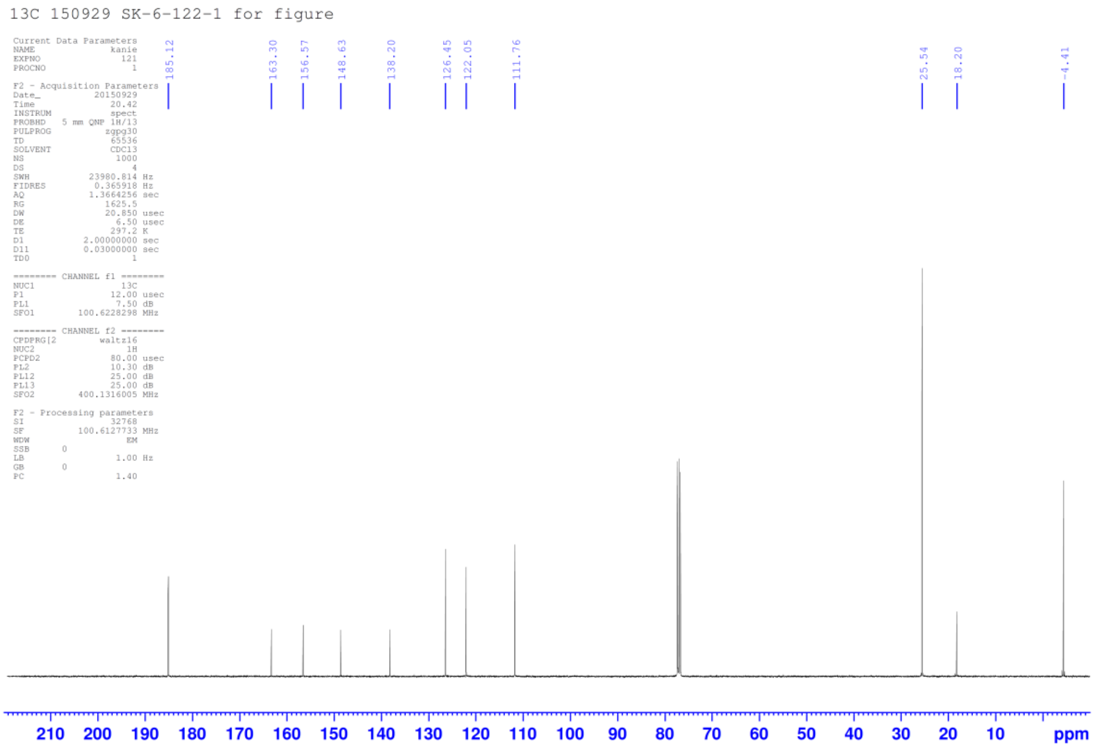
13C 150929 SK-6-122-1 for figure
185.12
163.30
156.57
148.63
138.20
126.45
122.05
111.76
25.54
18.20
-4.41
ppm

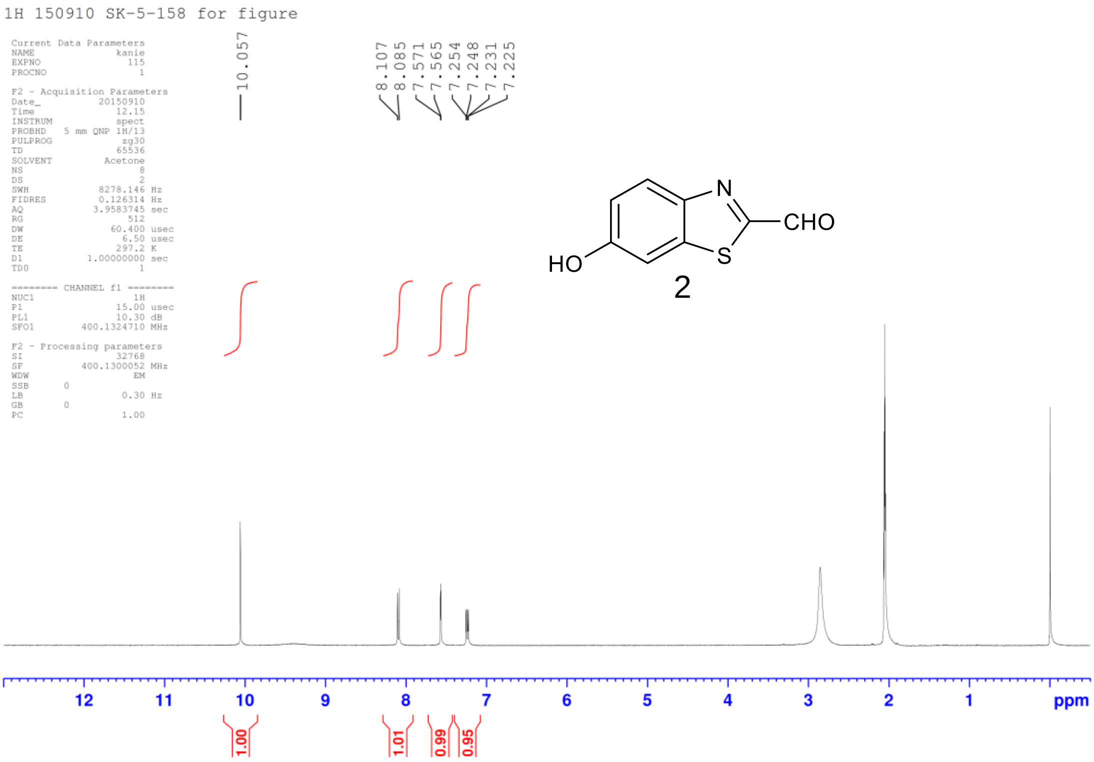
1H 150910 SK-5-158 for figure
Current Data Parameters
NAME kanie
EXPNO 115
PROCNO 1
F2 - Acquisition Parameters
Date_ 20150910
Time 12.15
INSTRUM spect
PROBHD 5 mm QNP 1H/13
PULPROG zg30
TD 65536
SOLVENT Acetone
NS 8
DS 2
SWH 8278.146 Hz
FIDRES 0.126314 Hz
AQ 3.9583745 sec
RG 512
DW 60.400 usec
DE 6.50 usec
TE 297.2 K
D1 1.00000000 sec
TD0 1
======== CHANNEL f1 ========
NUC1 1H
P1 15.00 usec
PL1 10.30 dB
SFO1 400.1324710 MHz
F2 - Processing parameters
SI 32768
SF 400.1300052 MHz
WDW EM
SSB 0
LB 0.30 Hz
GB 0
PC 1.00
10.057
8.107
8.085
7.571
7.565
7.254
7.248
7.231
7.225
N
S
CHO
HO
2
12
11
10
9
8
7
6
5
4
3
2
1
ppm
1.00
1.01
0.99
0.95

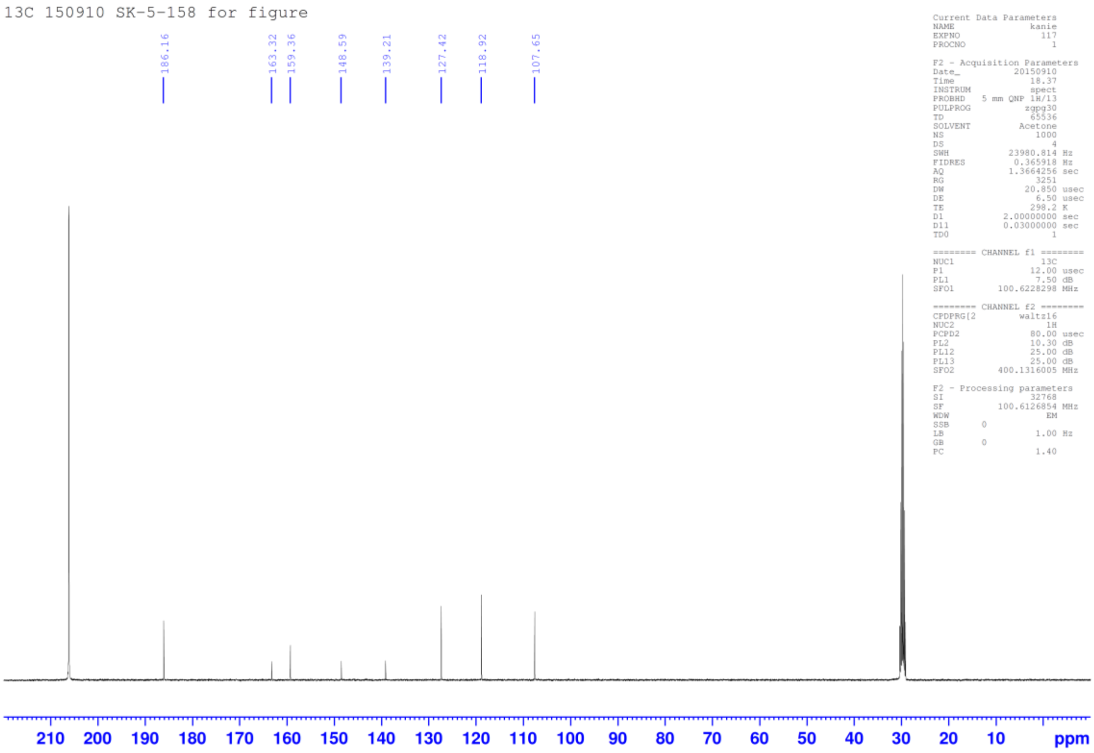
13C 150910 SK-5-158 for figure
186.16
163.32
159.36
148.59
139.21
127.42
118.92
107.65
Current Data Parameters
NAME kanie
EXPNO 117
PROCNO 1
F2 - Acquisition Parameters
Date_ 20150910
Time 18.37
INSTRUM spect
PROBHD 5 mm QNP 1H/13
PULPROG zgpg30
TD 65536
SOLVENT Acetone
NS 1000
DS 4
SWH 23980.814 Hz
FIDRES 0.365918 Hz
AQ 1.3664256 sec
RG 3251
DW 20.850 usec
DE 6.50 usec
TE 298.2 K
D1 2.00000000 sec
D11 0.03000000 sec
TD0 1
======== CHANNEL f1 ========
NUC1 13C
P1 12.00 usec
PL1 7.50 dB
SFO1 100.6228298 MHz
======== CHANNEL f2 ========
CPDPRG[2 waltz16
NUC2 1H
PCPD2 80.00 usec
PL2 10.30 dB
PL12 25.00 dB
PL13 25.00 dB
SFO2 400.1316005 MHz
F2 - Processing parameters
SI 32768
SF 100.6126854 MHz
WDW EM
SSB 0
LB 1.00 Hz
GB 0
PC 1.40
210
200
190
180
170
160
150
140
130
120
110
100
90
80
70
60
50
40
30
20
10
ppm
